# A Critical Analysis of Colour–Shape Correspondences: Examining the Replicability of Colour–Shape Associations

**DOI:** 10.1177/2041669519834042

**Published:** 2019-03-29

**Authors:** Noemi Dreksler, Charles Spence

**Affiliations:** Crossmodal Research Laboratory, Department of Experimental Psychology, University of Oxford, UK

**Keywords:** colour–shape correspondences, intramodal correspondences, replicability, colour, shape, emotional mediation, colour–shape associations

## Abstract

Research on the topic of colour–shape correspondences started in the early 20th century with the Bauhaus artist Wassily Kandinsky. However, more recently, the topic has been examined using the empirical framework of crossmodal correspondences research. The field remains one in which consistent results and generalisable hypotheses about the existence and nature of colour–shape correspondences are lacking. The replicability and consistency of findings concerning colour–shape correspondences are examined in three online colour–shape matching experiments using the same procedure and study design while varying the sets of shape stimuli that are evaluated. Participants matched one of 36 colours to each shape as well as made preference and arousal appraisal ratings for each of the shapes and colours. The complexities of analysing colour–shape correspondence data are discussed and illustrated by classifying and analysing shape and colours in a variety of different ways, including using continuous perceptual and objective measures. Significant colour–shape associations were found. However, as hypothesised, limited consistent results in regard to what perceptual shape characteristics predicted colour choices were documented across the three stimuli sets. This was the case both within and across different analysis methods. The factors that may be responsible for these inconsistencies are critically discussed. Intriguingly, however, evidence for emotional mediation, whereby shape and colour liking and arousal appraisals appear to influence the colour–shape correspondences made by participants, was found across all three experiments.

## Introduction

### The History of Colour–Shape Correspondences

Historical accounts of colour–shape correspondences usually begin with the Russian artist Wassily Kandinsky—a pioneer of modern abstract art and a key member of the Bauhaus collective. In search of a universal visual language, Kandinsky believed that lines, shapes, colours, and even music were not only essential to this endeavour, but fundamentally intertwined. Furthermore, he believed that a translation existed between graphical features (e.g., lines) and nongraphical dimensions such as colour, music, perception, emotions, and even spiritual intuition (Kandinsky, 1914, 1926/1994; Lupton & Miller, 1993). This notion of *translations* can be understood in many ways akin, at least in spirit, to the phenomenological and emotional associations between perceptual features and dimensions that have been documented in the case of crossmodal correspondences and, to a certain extent, in research on synaesthesia. Indeed, it has been suggested that Kandinsky may have been a synaesthete (Just, 2017, though see also Harrison, 2001).

**Figure 1.**
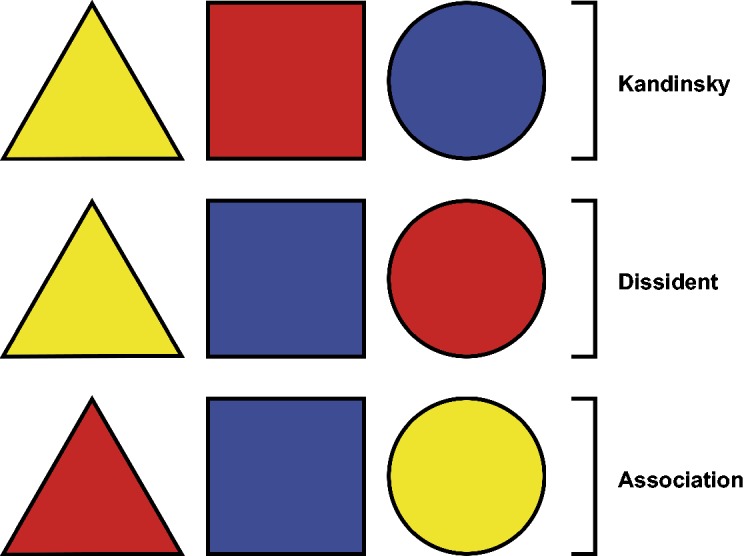
Kandinsky's colour–shape correspondences (yellow-triangle, red-square, blue-circle) together with two other configurations often found in more recent research on colour–shape correspondences. The dissident correspondences (yellow-triangle, blue-square, red-circle) are so named after those members of the Bauhaus movement who disagreed with Kandinsky's correspondences (see Gage, 1993). Chen et al. refer to them as the “Japanese colour–shape association[s]” (2015d, p. 2) or “the Japanese flag effect” (2015a, p. 5) because they consistently reappear in their research on Japanese participants. The association correspondences (red-triangle, blue-square, yellow-circle) are named after Jacobsen's (2002) suggestion that individuals choose combinations based on specific real-world object associations relevant to Jacobsen's German sample (e.g., sun—yellow circle, warning signal—red triangle; cf. Saluja & Stevenson, 2018).

The simplification of colour and form to primary shapes and colours was a key process for Kandinsky and other Bauhaus artists whose aim was to create an ideal means of communicating visually in the fields of both art and design (Jacobsen, 2002, 2004). In 1923, borrowing from the relevant experimental psychological methods of the time, Kandinsky famously handed out a questionnaire to workshop participants that involved his Bauhaus contemporaries answering which of three presented colours (red, blue, yellow) they would choose as the best match for a square, a triangle, and a circle. For Kandinsky, and a number of his contemporaries, a triangle was associated with yellow, a square was associated with red, and a circle was associated with blue (Gage, 1993). However, these associations have not always been chosen most frequently in recent research, which more commonly finds two other sets of associations, shown in Figure 1. Out of the six combinations possible for the Kandinsky colours and shapes, the dissident, association, and Kandinsky correspondences are the three most commonly chosen combinations across experiments (for a review of modern empirical findings on colour–shape correspondences, see Dreksler & Spence, 2019). But when colours are restricted to the three Kandinsky colours (e.g., Chen, Tanaka, & Watanabe, 2015d; Jacobsen, 2002), this also means that individuals may not be able to pick the colour that they truly feel best matches the shape.

### Modern Empirical Findings on Colour–Shape Correspondences

Jacobsen (2002) found the association correspondences to be the most commonly chosen and the Kandinsky correspondences to be one of the least often chosen. Jacobsen and Wolsdorff's (2007) study reported that the dissident, association, and Kandinsky correspondences were matched in that order with descending frequency. Meanwhile, Kharkhurin (2012, Experiment 2) found that not a single individual chose all three Kandinsky correspondences. Chen et al. (2015d) found, in one experiment, that the dissident correspondences (36.8%) and Kandinsky correspondences (36.8%) were chosen most commonly, while, in a second experiment, more than half of their participants chose the dissident correspondences (54.2%). All other associations were chosen less frequently (16.7% or less). Evidence from experiments using the implicit association test has been mixed (cf. Chen et al., 2015d; Makin & Wuerger, 2013; for more information on the use of the implicit association test as a measure of association, see Banaji & Greenwald, 2013; Parise & Spence, 2012), and Kharkhurin (2012) showed no evidence for Kandinsky's correspondences in a priming experiment.

Much of the research that has expanded beyond the three colours and three shapes of the Kandinsky correspondences has focused on 12 geometric shapes (triangle, square, circle, parallelogram, rhombus, hexagon, trapezium, oval, cone, pyramid, truncated cone, truncated pyramid; e.g., Chen, 2015; Chen et al., 2014; Chen, Tanaka, Matsuyoshi, & Watanabe, 2015a; Chen, Tanaka, Namatame, & Watanabe, 2015c, 2016). Each shape is presented in the middle of a colour wheel—a set of 40 hues from the Natural Colour System, which are then divided into eight categories—blue, red, yellow, green, yellow/green, blue/red or purple, yellow/red or orange, blue/green—for the sake of analysis (see Malfatti, 2014, p. 18). The participant chooses the colour from the wheel that they feel best matches the shape in the centre. Across the four experiments of this type conducted by two different research groups, the triangle was consistently associated with the colour category ‘yellow’ and the circle with the category ‘red’ (Chen et al., 2014, 2015a, 2015c; Malfatti, 2014, reported in Albertazzi et al., 2013). According to researchers such as Chen et al. (2015a) and Albertazzi et al. (2013), certain shape features may be associated with different colour temperatures.^1^ Overall, in the studies reported by these researchers, there appears to be a trend towards shapes based on the triangle and acute angles (rhombus, pyramid) being associated with yellow. Shapes that are not curved and include right or obtuse angles (e.g., square, hexagon, parallelogram, and truncated pyramid), mapped onto colder hues more consistently. Shapes consisting of curved lines, by contrast, were associated with warmer hues (circle, cone, oval). Testing for this effect of angles specifically, Malfatti (2014, Experiment 3, also reported in Albertazzi, Malfatti, Canal, & Micciolo, 2015) reported that acute angles were associated with warmer colours, while obtuse angles were associated with colder colours instead.

Malfatti's doctoral work alone attempted to expand the stimulus set in a way that systematically varied shapes (open/closed lines, symmetry, number of generating points, pointy/rounded) that individuals had to match to a wide array of colours (37 Berkeley Colour Project colours). Malfatti found that pointedness, complexity, and symmetry were important perceptual drivers of the saturation, lightness, red/greenness, and yellow/blueness of the colours that were chosen to match the shapes. But the way in which these factors were related was stimulus dependent (i.e., not consistent across the two types of shape stimuli sets—open line shapes and closed geometric shapes—presented to individuals). It is therefore obviously difficult to draw any general conclusions as to what shape features may drive colour choices, especially when no one has, at least not as far as we are aware, attempted to replicate this work until now. In addition, Malfatti uses a different method than all previous research, whereby individuals chose the three colours that they found most consistent and the three they considered most inconsistent with the presented shape. A statistical shape–colour association (SCA) score can then be calculated for each colour dimension based on the three most consistent and inconsistent choices.

To date, few researchers have attempted to grapple with the inconsistencies that have been reported across experiments and research groups in an in-depth manner. In fact, many of the introductions to this topic fail to mention these (e.g., Chen et al., 2014, 2015a, 2015c) beyond calling into question the Kandinsky correspondences (e.g., Kharkhurin, 2012), thus making it difficult for a reader of the literature to gain a realistic overview of the state of the field. What is clear is that it is an open question as to whether generalisable conclusions can be drawn about what perceptual features (e.g., perceptually rated dimension such as ‘pointedness’) may drive colour–shape associations and whether there is a consensus across samples of correspondences between specific shapes and colours that can be replicated reliably.

### Intramodal and Crossmodal Correspondences

Strictly speaking, colour–shape correspondences are intramodal, at least as they are commonly assessed (namely by viewing shapes rather than, for instance, feeling them^2^; for discussion of crossmodal correspondences involving shape evaluated in the tactile dimension for the case of sound symbolism, see Fryer, Freeman, & Pring, 2014). But much of the modern empirical work in this area, described earlier, has typically been grounded in the field of crossmodal correspondences research: that is, the bidirectional (i.e., transitive), nonarbitrary mappings between the attributes (or dimensions) of two sensory modalities, which can give rise to congruency effects in performance and are usually considered to match one another phenomenologically (Parise & Spence, 2013; Spence, 2011). Crossmodal and colour–shape correspondences are by no means a new field of study (e.g., Köhler, 1929, 1947). And, in recent years, the study of crossmodal correspondences has seen a large upswing and developed into a fully fledged field of multisensory research in its own right (e.g. see Parise, Spence, & Deroy, 2016).

Furthermore, it has recently been argued that crossmodal correspondences could be a fundamental feature of multisensory perception (i.e., the integration of unisensory signals) and can perhaps be considered a likely third candidate alongside spatiotemporal and semantic congruency when it comes to solving the crossmodal binding problem (Ernst, 2007; Spence, 2011). Intramodal correspondences and how they may relate to, and differ from, crossmodal correspondences remain unexplored areas, but much of the way we think about colour–shape correspondences is shaped by the field of crossmodal correspondences research. As such, investigating colour–shape correspondences can also tell us more about how different intramodal aspects of our senses are interlinked and, similarly to crossmodal correspondences, could have applied effects in the field of design and aesthetics (e.g., Ngo, Piqueras-Fiszman, & Spence, 2012; Spence, 2012; Velasco, Woods, Petit, Cheok, & Spence, 2016).

### Causal Mechanisms of Colour–Shape Correspondences

Spence et al. have suggested four explanations that might underlie the acquisition and nature of *crossmodal*^3^ correspondences: affective, semantic, statistical, and structural (Parise & Spence, 2013; Spence, 2011, 2018). Of course, these are not mutually exclusive—any correspondence could find its causal origins in more than one of these explanations:

1. Affective correspondences would be acquired through the association of two dimensions based on similar valences of emotions, pleasantness, or hedonic responses.
2. Semantic correspondences are linguistically or conceptually mediated—associated features are linked because they are described using a similar semantic dimension and common label (e.g., weak/strong).
3. Statistical correspondences depend on sensory and memory systems tracking the regularities in the patterns of sensory signals we receive through experience.
4. Structural correspondences are either innate or exist due to the maturating of sensory coding structures across development and are posited to depend on common coding systems (e.g. for stimulus intensity or magnitude) as well as interaction effects resulting from neural connections between sensory processing areas.

Malfatti (2014) has explored what causal mechanisms could underlie colour–shape correspondences by exploring affective associations. By using Osgood's semantic differential technique (Osgood, Suci, & Tannenbaum, 1957), Malfatti found that both emotional (e.g., harmfulness, strength, anger) and nonemotional conceptual dimensions (e.g., pleasantness, preference)^4^ associated with a shape and the three most consistently chosen and the three most inconsistently chosen colours exhibited large statistically significant correlations (e.g., for closed line shapes, anger, *r* = .62; harmfulness, *r* = .68; activity, *r* = .46; liking, *r* = .57; pleasantness, *r* = .57). Overall, individuals tended to choose colours they liked for shapes they liked, as well as colours more emotionally consistent in their associations with shapes that aligned with these emotional associations (also see Chen, Tanaka, Matsuyoshi, & Watanabe, 2015b for evidence that there may be cross preferences for colour and shape features among individuals).

### Complexities Associated With Analysing Colour and Shape

Compared with some other dimensions explored to date in crossmodal correspondences research (e.g., taste, simple tones, 2D visual space), unisensory colour–shape correspondence research is made all the more complex by the many ways in which both colours and shapes can be characterised and analysed (see Table 1, although of course some crossmodal correspondences, such as smell–shape or smell–colour correspondences, run into similar complexity problems in terms of the stimuli). This is further complicated by the fact that many of the features of colours and shapes cannot be cleanly defined as prothetic dimensions that are organised as more or less than any other point on the dimension scale (e.g., loudness, brightness) and are instead conceptualised as metathetic features where the change in stimulus is a change in quality rather than in quantity (see Stevens, 1957; though for early discussion that red saturation at least could be classed as a prothetic dimension, see Panek & Stevens, 1966, and Gilbert, Fridlund, & Lucchina, 2016, supplementary material, for how hue can be statistically analysed in a circular fashion). Both the breadth of analysable features and, in some cases, their inherent metathetic nature can make comparison across features difficult, especially in the case of inconsistent results across examined features. Malfatti (2014), for example, found variance in lightness in closed shapes was predicted by geometrically determined symmetry axes (8%), pointedness (11%), and concavities (12%) but was associated only with complexity (23%) when taking into account perceptual measures (i.e., like those measures denoted by [P] in Table 1, rather than more objective [O] shape characteristics).

**Table 1.** Variety of Ways in Which Colour and Shape Features Can Be Categorised, Rated, and Perceived.

	Perceptual [P]	Objective [O]
Colour	Red/Green Yellow/Blue Red Green Yellow Blue Lightness Saturation Warmth	RGB Red, RGB Green, RGB Blue CMYK Cyan, CMYK Magenta, CMYK Yellow, CMYK Black HSL Hue, HSL Saturation, HSL Lightness LAB Lightness, LAB Green/Red, LAB Blue/Yellow
Shape	Roundedness Symmetry Complexity	Symmetry by category Symmetry axes Concavities Generating points Number of sides Complexity by levels Roundedness by category

*Note*. Here, and in the rest of the article, colour and shape features rated by individuals are referred to as perceptual and denoted by a [P]. These are usually rated on a polar dimension rating scale (e.g., redness may be rated on a scale from *not at all red* to *very red*, while complexity may be rated on a scale from simple – complex). Alongside these perceptual qualities, shapes and colours can be classified using more objective measures [O], such as levels or categories the researcher has assigned to shapes based on their observable qualities and the method by which they were designed. Objective colour information is determined by formal colour systems. It should be noted that objective colour information has not been used in previous research, and our focus overall will be on perceptual measures of colour for the sake of brevity as we compare results across three experiments. That is to say, colours are rated on various perceptual dimensions (e.g., lightness, light-dark) by a group of participants, instead of, for example, the HSL colour system's lightness value being used as an objective measure.

### The Present Research

Previous research findings on colour–shape correspondences are incredibly mixed and complex, and the results are rarely replicated reliably and consistently outside of specific research groups (see Dreksler & Spence, 2019 for an extensive review of prior colour–shape correspondence research). As such, the objective of the following three online experiments (see Rothen, Seth, Witzel, & Ward, 2013; Witthoft, Winawer, & Eagleman, 2015 for examples of previous colour-related multisensory research using online methodologies) was to focus on what results might be replicable across three experiments using the exact same design and procedure but presenting three different sets of stimuli to three cross-cultural samples. To do this, a variety of statistical methods and ways of ‘slicing’ both shape and colour data will be used to demonstrate the complexity of analysing colour–shape correspondences so that future research can begin to tackle the following three questions more effectively:

1. Are there colour–shape correspondences between specific shapes and hues?
2. Do specific shape characteristics predict the colours that are chosen to best match them, and do they do so across different types of shape stimuli, experiments, and research groups?
3. To what extent can the emotional mediation hypothesis (Palmer, Schloss, Xu, & Prado-León, 2013) account for colour–shape matching behaviour? That is to say, is there a correlation between the hedonic appraisals individuals make for a shape and the hedonic appraisals of the colour they choose that best matches the shape?

In regard to the first two questions, based on previous research (cf. closed vs. open line shape results in Malfatti, 2014), we expected to find significant colour–shape association effects within each experiment but that these may be highly stimulus dependent and as such do not replicate consistently across the three experiments. Similarly, initial evidence from Malfatti suggests that emotional mediation effects are likely to be found, as they have been for other crossmodal correspondences involving colour (e.g., Gilbert et al., 2016; Palmer et al., 2013; Schifferstein & Tanudjaja, 2004) and, as such, predicted that they would be present more consistently across the three experiments and three stimuli sets.

## Method

### Participants

Three experiments were conducted to examine colour–shape correspondences. Participants were excluded if they reported (a) noncorrected vision impairments that would hinder perceiving the colour and shape stimuli correctly (including making a response to the City University colour blindness test that indicated some sort of colour deficiency); (b) that they experienced synaesthesia related to colours, shapes, and graphemes; and (c) exhibited repetitive responding patterns indicative of a failure to read and answer the questions properly (e.g., responding 100 on a large number of rating scales in a row).

A cross-cultural sample of participants was recruited online on Prolific (https://prolific.ac) with the requirement that participants be fluent in English, having a more than 98% acceptance rates on the platform to exclude fraudulent users and bots, and not having participated in any of the first author's previously conducted experiments related to colour or shape. Sixty-four participants took part in Experiment 1 (27 identified as female, mean age = 30.33 years, *SD* = 9.80), 68 participants participated in Experiment 2 (39 identified as female, 1 as agender, mean age = 31.41 years, *SD* = 9.69), and 75 participants participated in Experiment 3 (49 identified as female, mean age = 32.59 years, *SD* = 9.95). As highlighted by the mean ages of participants, one benefit of conducting this research online is gaining access to a broader sample of individuals than the undergraduate university students that much of psychological research is based on (Henrich, Heine, & Norenzayan, 2010; for a review of the representativeness and use of online samples for perception research, see Woods, Velasco, Levitan, Wan, & Spence, 2015).

### Materials

**Figure 2.**
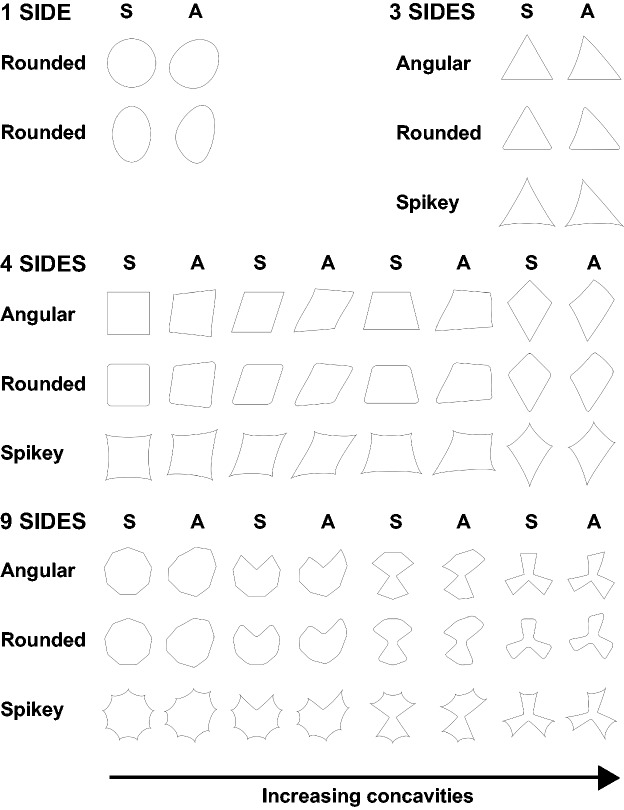
The 58 geometric shapes presented in Experiment 1. Columns labelled ‘S’ are symmetrical shapes, and columns labelled ‘A’ are asymmetrical shapes.

Fifty-eight geometric shapes (see Figure 2) were designed for use in Experiment 1 using Adobe Illustrator. They were designed to vary by roundedness (three levels: angular/rounded/spikey), symmetry (two levels: symmetrical/asymmetrical), and complexity. Complexity was varied by varying the number of sides (four levels: 1, 3, 4, 9) and levels of concavities (four levels: 0, 1, 2, 3) of the shape. These shapes are similar to those used by Malfatti (2014) and are closest to the geometric shapes discussed by Kandinsky (1914) and the shape stimuli used in studies by Chen et al. (2014, 2015a, 2015c).

**Figure 3.**
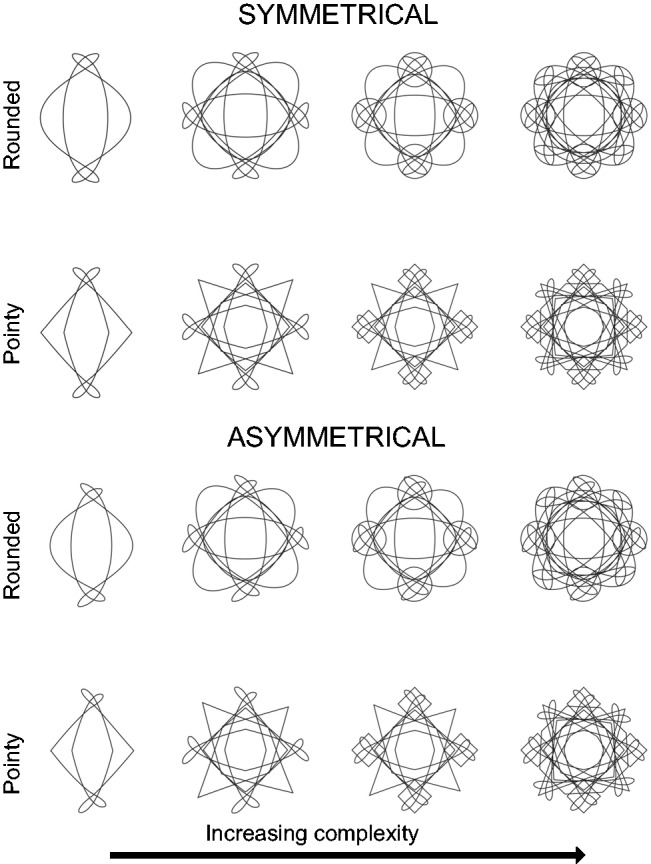
The 16 mandala shapes presented in Experiment 2.

Sixteen mandala shapes (see Figure 3) were created for use in Experiment 2 using Adobe Illustrator and were designed to vary by roundedness (two levels: rounded/pointy), symmetry (two levels: symmetrical/asymmetrical), and complexity (four levels of increasing complexity created by adding additional repeating elements).

**Figure 4.**
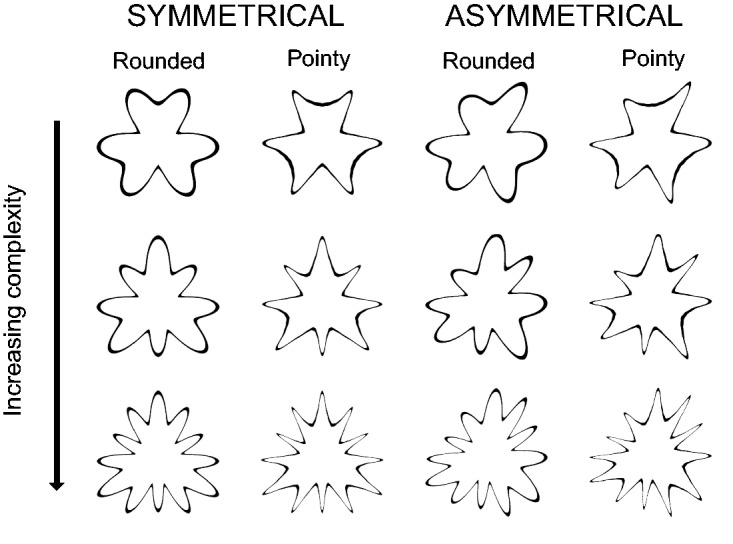
The 12 RF shapes presented in Experiment 3.

Twelve radial frequency (RF) shapes (see Figure 4) were generated using the MATLAB code of Pi-Chun Huang (see online documentation of Turoman, Velasco, Chen, Huang, & Spence, 2018) and are most akin to the traditional Bouba/Kiki shapes used in sound symbolism research (Maurer, Pathman, & Mondloch, 2006; see Chen, Huang, Woods, & Spence, 2016, for an example of how RF pattern shapes can be used in sound symbolism/crossmodal correspondences research). Furthermore, they tie in with other crossmodal correspondence work that has explored the relationship of shape and taste as well as shape and odour (e.g., Hanson-Vaux, Crisinel, & Spence, 2012; Salgado-Montejo et al., 2015). They were designed to vary by roundedness (two levels: rounded/pointy), symmetry (two levels: symmetrical/asymmetrical), and complexity (three levels of increasing complexity created by adding extra protruding points on each shape).

### Design and Procedure

The same design and procedure were used for all three experiments to facilitate the examination of replicability across studies of colour–shape correspondence effects. All three experiments were conducted using an online Qualtrics questionnaire that participants accessed through the participant recruitment platform Prolific. Participants provided their Prolific ID and were then requested to sit in front of their computer, set so that the stimuli could be viewed easily, in a normally lit room that was quiet and free from distractions. They then proceeded to give their informed consent to participate in the study. An outline of the structure and instructions for each section of the study were presented to the participants.

**Figure 5.**
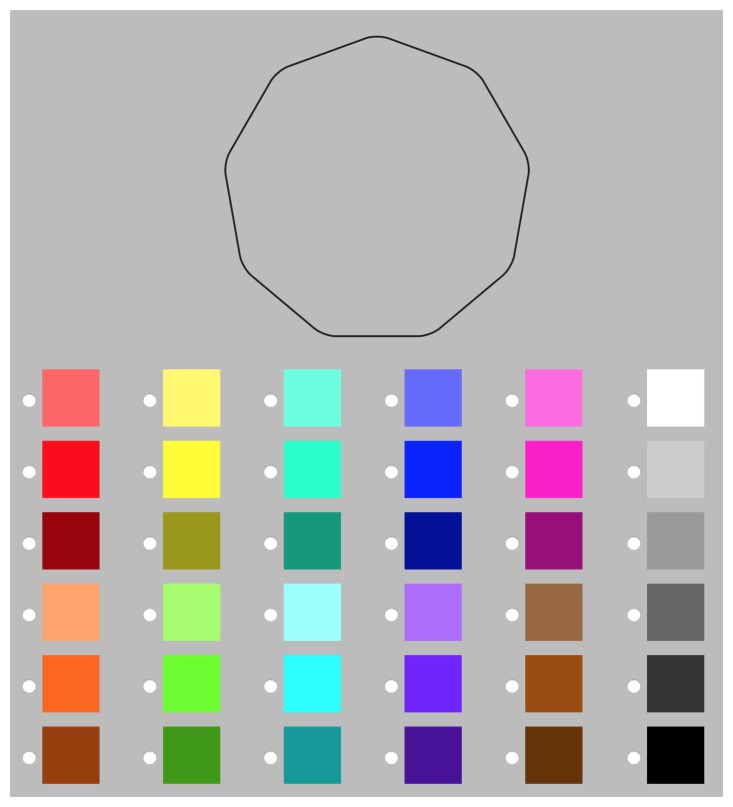
The way in which the shape and colours were presented to participants during the colour–shape matching task block with nonrandomised colours. The 36 colours were chosen to cover a breadth of hues (in descending rows of three, column by column: red, orange, yellow, green, aquamarine, sky, blue, purple, pink, brown, white/grey, grey/black), presented both at full saturation (rows 2 and 5) and at different levels of lightness (rows 1 and 4: lighter than the fully saturated second row; rows 3 and 6: darker than the fully saturated fifth row). All shapes and colours throughout the experiment were presented on a grey background (#BCBCBC).

Participants were randomly and evenly allocated to one of two colour–shape matching task blocks where they were presented with the following instructions: ‘For each shape please choose the colour that you think best matches it. There are no right or wrong answers. Please answer as intuitively as possible.’ For each shape, the participant chose one of the 36 colours and rated how intuitive they found their preceding match on a scale of ‘I chose completely randomly’ to ‘This match feels very right.’ This was included to explore individual differences in experience of the task and each individual match. In one colour–shape matching block, the colours were presented in the same order (see Figure 5) as is common in colour–shape correspondence research, while in the other, colours were presented in a randomised fashion. The data were collapsed across these two block conditions for all three experiments due to lack of significant differences in the perceptual colour characteristics chosen in the two blocks.

After participants had chosen a colour for each shape, they were presented with four blocks in a random order, and they were asked to rate the valence (very unpleasant–very pleasant) and arousal (not at all arousing–very arousing) of all the colours and shapes in the study. To avoid any lexical ambiguity and confusion, the participants were reminded that ‘arousing is meant in a nonsexual sense, it refers to a general state of activation and energy.’

In the final section of the study, the participants completed an array of psychometrics and individual difference questions including the Big Five Inventory (John, Naumann, & Soto, 2008), 20-item Toronto Alexithymia Scale (measures alexithymia; Bagby, Parker, & Taylor, 1994), Vividness of Visual Imagery Questionnaire (measures visual mental imagery; Marks, 1973), and Autism Spectrum Quotient (Baron-Cohen, Wheelwright, Skinner, Martin, & Clubley, 2001), questions regarding their age, gender, and cultural background, whether they experienced synaesthesia, and the City University online colour-blindness test (‘A New Web-Based Colour Vision Test,’ n.d.). Personality psychometrics are not discussed in this article because they are not key to the discussion of replicability of colour–shape correspondence findings and do not appear to explain any variance between individuals (for a full discussion of the role of individual differences in colour–shape correspondences, see Dreksler, 2019). Upon finishing, the participants were presented with a completion code and URL and referred back to Prolific.

### Additional Data

Additional surveys were conducted to analyse the three experiments using Qualtrics surveys with participants recruited online through Prolific. Forty-one participants were asked to rate the perceptual ([P]) roundedness (rounded–pointy), symmetry (asymmetric–symmetric), and complexity (simple–complex) of the mandala and RF shapes presented in Experiments 2 and 3 on polar dimension rating scales, and a further 25 participants completed the same ratings for the 58 shapes used in Experiment 1. Another 36 participants rated the 36 colours on 9 perceptual ([P]) colour dimensions: red/green (red–green), yellow/blue (yellow–blue),^5^ red (not all red–very red), green (not at all green–very green), yellow (not at all yellow–very yellow), blue (not at all blue–very blue), lightness (light–dark), saturation (unsaturated–saturated), and warmth (cold–warm).

### Data Restructuring

To analyse the data, it was formatted into two structures:

1. Case-by-case data where each row represents one choice made by a participant and the columns contain all of the information on colour choices and their corresponding perceptual and objective information, valence and arousal ratings, and individual differences;
2. SCA data where each row represents one shape and the columns correspond to the average perceptual colour feature associated with the colours chosen by individuals for that shape (e.g., red [P]) and perceptual and objective shape information, such as average perceptual shape ratings (e.g., complexity [P]) and the shape's corresponding shape categories and levels (e.g., symmetry [O] coded as the two levels of symmetrical and asymmetrical).

While the aggregate approach akin to the SCA analysis is most common in research on the crossmodal correspondences, it is arguably also worth considering case-by-case data as well (e.g., Reinoso Carvalho et al., 2015). Because perceptual colour–shape association analyses depend on consensual values of the perceptual characteristics of both the shapes and colours, SCA data alongside case-by-case data may be more applicable for investigating such perceptual effects. Because we cannot necessarily assume that hedonic appraisals are consistent across participants, it is especially in these analyses that case-by-case data may be particularly relevant and enlightening.

All analyses were conducted using IBM SPSS Statistics version 24.

## Results

### Colour and Shape Associations—Contingency Tables and Chi-Square Analyses

One of the most common ways that many colour–shape correspondence studies have evaluated whether there are significant colour–shape associations is through chi-square analyses of the categorical factors of shape and colour categories. To avoid cell counts that may be too small during chi-square analyses, the thirty-six colours were grouped into 12 triplets by colour hue as described in Figure 5 (red, orange, yellow, green, aquamarine, sky, blue, purple, pink, brown, white/grey, grey/black). Pearson chi-square analyses were conducted to determine whether colour and shape were significantly associated in each of the three experiments. Colour and shape revealed significant associations in both Experiments 1 (χ^2^ = 1052, *df* = 627, *p* < .001) and 3 (χ^2^ = 226, *df* = 121, *p* < .001), but not for Experiment 2.

A residual analysis was conducted to determine whether the count in each cell was significantly higher or lower than expected. Tables 2 and 3 represent the cross-tabulation contingency row profiles for Experiments 1 and 3, which both showed significant colour–shape associations in the chi-square analyses. Each cell indicates the percentage of individuals choosing the corresponding column's colour for each row's shape. When the adjusted standardised residual for each cell was greater than 2, indicating this colour was chosen more frequently than expected, this is indicated by bold type. If the adjusted standardised residual for each cell was less than 2, indicating this colour was chosen less frequently than expected, this is indicated by underlined type.

**Table 2.** A Contingency Table Showing the Percentage of Individuals Choosing Each Colour Triplet for Each Shape in Experiment 1 and the Positive (Bold) and Negative (Underlined) Adjusted Standardised Residuals of Those Cells Greater Than 2 and Less Than −2, Respectively.

		Red	Orange	Yellow	Green	Aquamarine	Sky	Blue	Purple	Pink	Brown	White-Grey	Grey-Black
	Shape												
1	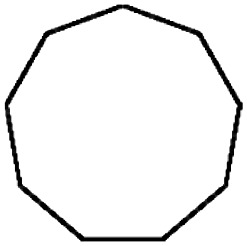	17.2	3.1	7.8	6.3	6.3	9.4	**15.6**	4.7	6.3	9.4	6.3	7.8
2	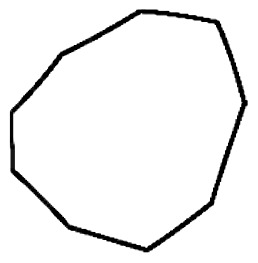	3.1	4.7	12.5	6.3	3.1	7.8	7.8	9.4	6.3	6.3	20.3	12.5
3	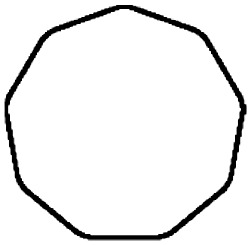	**23.4**	1.6	4.7	6.3	6.3	7.8	6.3	7.8	**15.6**	6.3	9.4	4.7
4	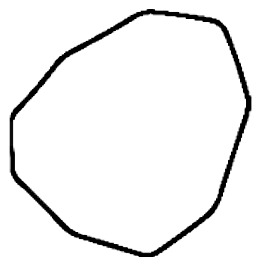	3.1	9.4	7.8	9.4	0	3.1	9.4	10.9	3.1	**14.1**	**25.0**	4.7
5	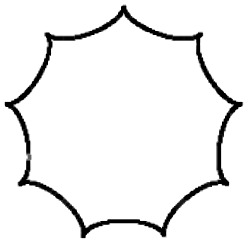	7.8	1.6	**34.4**	7.8	6.3	9.4	4.7	3.1	6.3	1.6	9.4	7.8
6	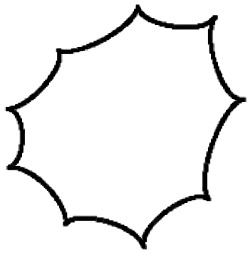	15.6	3.1	**23.4**	9.4	4.7	3.1	3.1	4.7	7.8	1.6	10.9	12.5
7	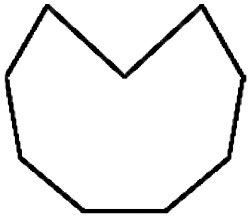	4.7	6.3	**35.9**	3.1	3.1	6.3	7.8	0	3.1	6.3	9.4	14.1
8	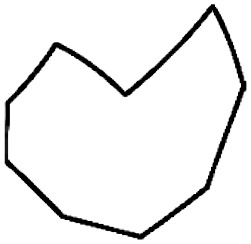	10.9	9.4	**25.0**	9.4	4.7	7.8	4.7	6.3	4.7	6.3	6.3	4.7
9	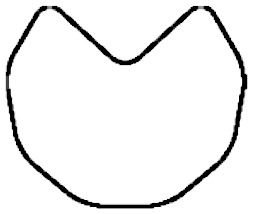	7.8	3.1	**34.4**	7.8	0	6.3	6.3	6.3	9.4	6.3	6.3	6.3
10	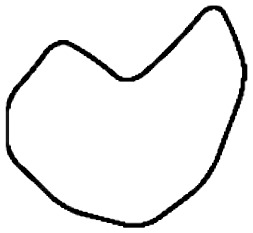	14.1	4.7	18.8	6.3	7.8	9.4	4.7	3.1	9.4	**14.1**	4.7	3.1
11	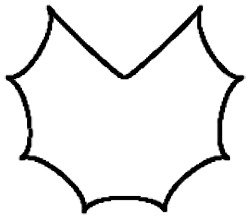	3.1	1.6	10.9	4.7	3.1	0	10.9	10.9	7.8	3.1	4.7	**39.1**
12	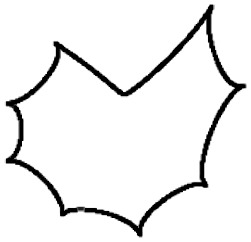	7.8	6.3	**25.0**	6.3	1.6	1.6	9.4	4.7	9.4	6.3	7.8	14.1
13	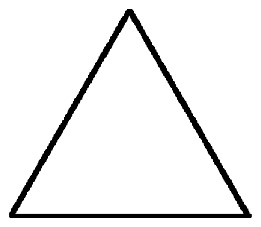	**28.1**	6.3	**20.3**	7.8	1.6	0	6.3	12.5	3.1	3.1	7.8	3.1
14	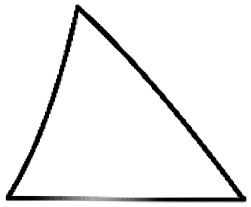	14.1	6.3	9.4	9.4	**12.5**	4.7	12.5	3.1	0	3.1	17.2	7.8
15	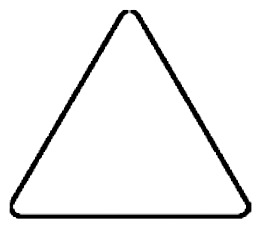	**32.8**	6.3	**21.9**	7.8	0	4.7	12.5	3.1	1.6	3.1	3.1	3.1
16	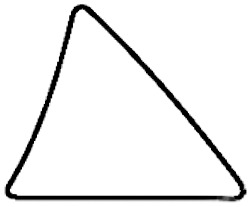	12.5	6.3	12.5	7.8	3.1	9.4	14.1	1.6	4.7	3.1	17.2	7.8
17	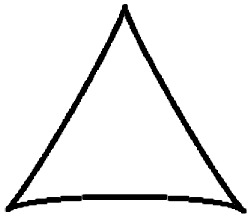	17.2	3.1	15.6	10.9	4.7	6.3	4.7	10.9	9.4	9.4	4.7	3.1
18	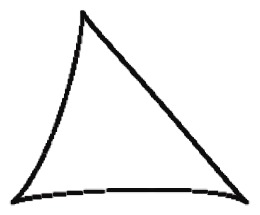	7.8	9.4	14.1	12.5	6.3	6.3	**17.2**	4.7	3.1	3.1	14.1	1.6
19	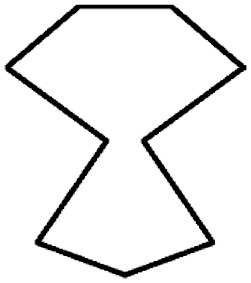	7.8	9.4	14.1	10.9	3.1	6.3	6.3	3.1	**15.6**	7.8	7.8	7.8
20	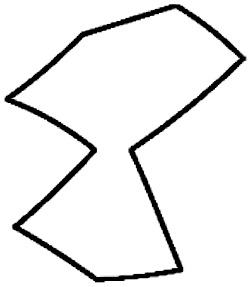	7.8	6.3	9.4	7.8	4.7	10.9	6.3	7.8	10.9	10.9	6.3	10.9
21	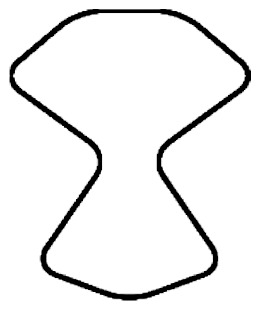	6.3	7.8	10.9	7.8	**12.5**	10.9	3.1	7.8	10.9	6.3	9.4	6.3
22	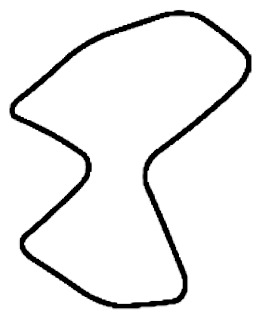	4.7	6.3	12.5	12.5	6.3	7.8	6.3	9.4	9.4	7.8	10.9	6.3
23	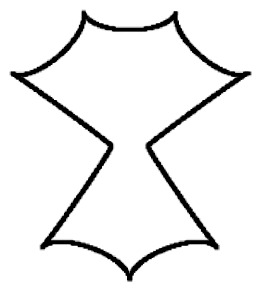	10.9	12.5	14.1	9.4	3.1	0	7.8	3.1	10.9	6.3	7.8	14.1
24	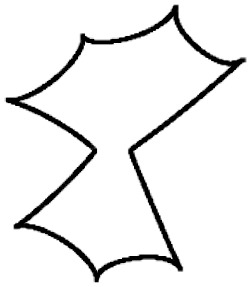	10.9	9.4	12.5	**15.6**	9.4	9.4	6.3	3.1	4.7	3.1	7.8	7.8
25	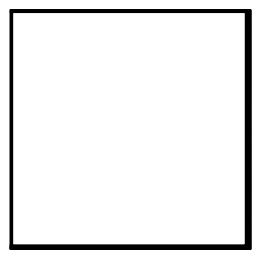	17.2	7.8	9.4	9.4	1.6	3.1	12.5	4.7	6.3	3.1	12.5	12.5
26	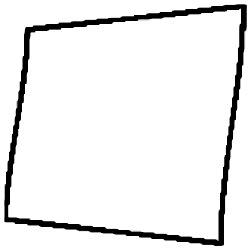	7.8	7.8	3.1	7.8	3.1	9.4	6.3	9.4	9.4	6.3	**21.9**	7.8
27	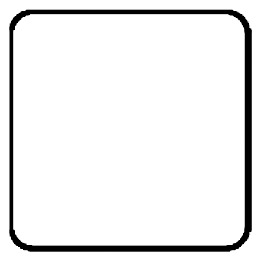	15.6	6.3	3.1	7.8	4.7	4.7	**17.2**	6.3	6.3	1.6	12.5	14.1
28	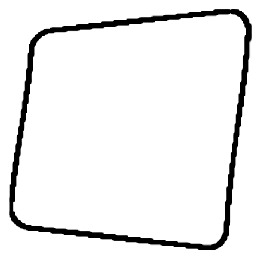	4.7	7.8	4.7	7.8	9.4	12.5	**17.2**	6.3	4.7	4.7	10.9	9.4
29	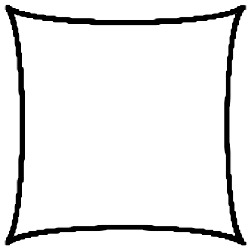	14.1	6.3	10.9	9.4	1.6	4.7	4.7	10.9	4.7	7.8	18.8	6.3
30	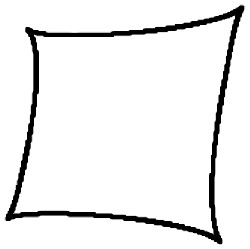	17.2	4.7	3.1	7.8	7.8	7.8	9.4	6.3	4.7	6.3	18.8	6.3
31	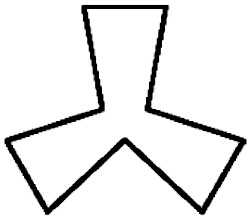	14.1	6.3	6.3	10.9	6.3	1.6	7.8	3.1	3.1	3.1	14.1	**23.4**
32	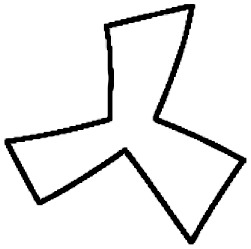	17.2	9.4	4.7	9.4	6.3	3.1	7.8	7.8	7.8	3.1	9.4	14.1
33	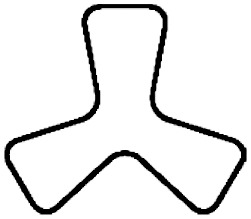	14.1	6.3	6.3	12.5	7.8	6.3	9.4	9.4	4.7	1.6	10.9	10.9
34	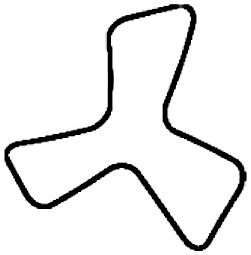	10.9	3.1	7.8	9.4	9.4	7.8	7.8	10.9	9.4	4.7	9.4	9.4
35	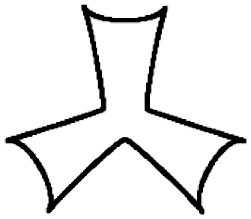	17.2	6.3	6.3	7.8	9.4	4.7	3.1	6.3	6.3	6.3	12.5	14.1
36	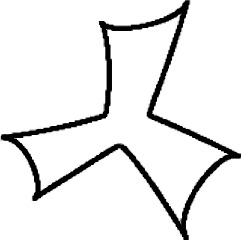	15.6	3.1	9.4	7.8	9.4	6.3	7.8	3.1	6.3	6.3	12.5	12.5
37	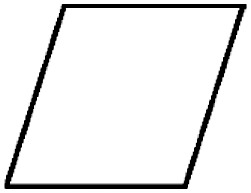	7.8	4.7	4.7	9.4	6.3	6.3	**18.8**	3.1	4.7	7.8	14.1	12.5
38	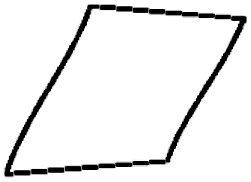	6.3	9.4	7.8	7.8	1.6	7.8	9.4	10.9	6.3	7.8	**23.4**	1.6
39	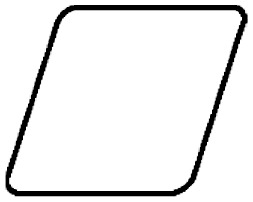	15.6	6.3	3.1	7.8	10.9	9.4	6.3	4.7	7.8	4.7	14.1	9.4
40	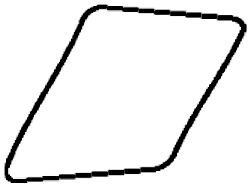	7.8	7.8	9.4	7.8	4.7	12.5	6.3	7.8	3.1	1.6	20.3	10.9
41	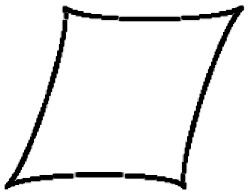	10.9	6.3	6.3	7.8	7.8	12.5	10.9	4.7	9.4	7.8	12.5	3.1
42	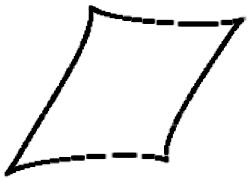	14.1	6.3	6.3	6.3	3.1	7.8	9.4	4.7	10.9	6.3	17.2	7.8
43	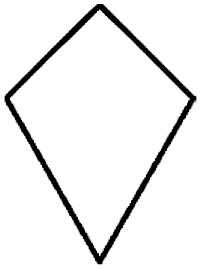	**20.3**	3.1	10.9	6.3	7.8	7.8	4.7	4.7	**15.6**	4.7	9.4	4.7
44	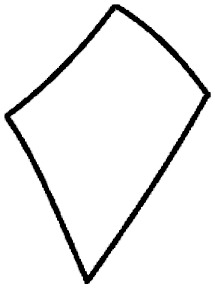	18.8	7.8	9.4	10.9	7.8	4.7	6.3	3.1	9.4	4.7	15.6	1.6
45	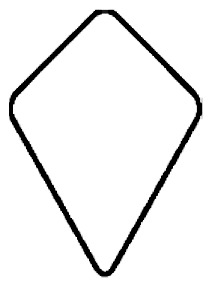	12.5	4.7	9.4	4.7	7.8	10.9	7.8	3.1	**15.6**	6.3	14.1	3.1
46	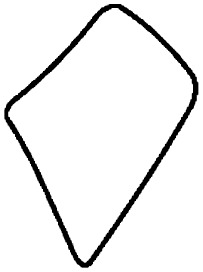	14.1	9.4	9.4	9.4	9.4	7.8	9.4	3.1	9.4	9.4	7.8	1.6
47	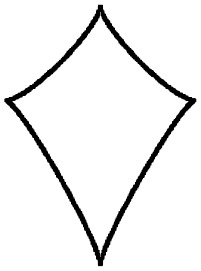	**20.3**	6.3	12.5	6.3	3.1	12.5	4.7	7.8	10.9	1.6	7.8	6.3
48	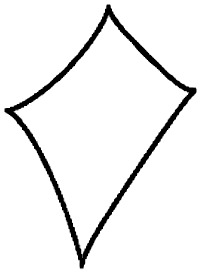	14.1	4.7	14.1	1.6	6.3	7.8	9.4	9.4	**14.1**	0	15.6	3.1
49	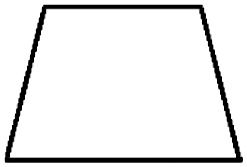	**20.3**	6.3	6.3	12.5	0	4.7	10.9	9.4	4.7	6.3	12.5	6.3
50	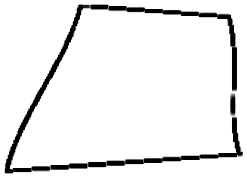	4.7	7.8	10.9	6.3	9.4	9.4	12.5	7.8	4.7	3.1	15.6	7.8
51	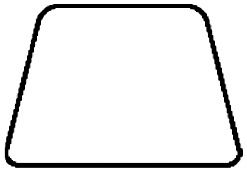	10.9	4.7	1.6	9.4	6.3	10.9	10.9	**12.5**	0	10.9	9.4	12.5
52	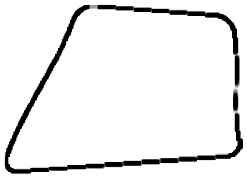	10.9	10.9	7.8	4.7	9.4	9.4	12.5	9.4	9.4	4.7	9.4	1.6
53	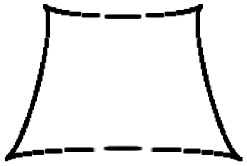	4.7	3.1	9.4	6.3	10.9	**15.6**	9.4	4.7	4.7	4.7	17.2	9.4
54	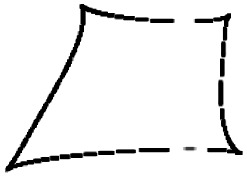	14.1	10.9	10.9	7.8	0	9.4	7.8	**15.6**	6.3	4.7	10.9	1.6
55	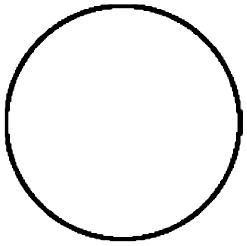	**20.3**	3.1	**23.4**	0	0	3.1	12.5	0	6.3	4.7	18.8	7.8
56	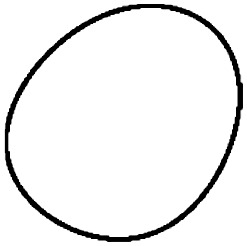	7.8	10.9	4.7	9.4	6.3	9.4	4.7	3.1	6.3	10.9	**23.4**	3.1
57	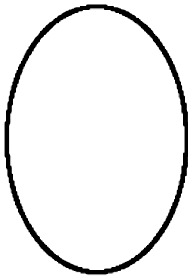	3.1	**17.2**	17.2	6.3	4.7	1.6	3.1	7.8	6.3	6.3	**25.0**	1.6
58	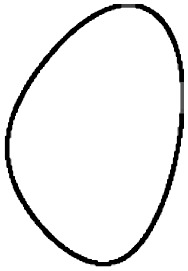	3.1	12.5	15.6	6.3	9.4	6.3	4.7	1.6	1.6	**12.5**	**23.4**	3.1
	Average	12.2	6.6	12.0	8.0	5.6	7.0	8.6	6.3	7.1	5.8	12.7	8.1

*Note*. The ‘average’ row represents the average percentage of individuals choosing each colour category across all shapes.

**Table 3.** A Contingency Table Showing the Percentage of Individuals Choosing Each Colour Triplet for Each Shape in Experiment 3 and the Positive (Bold) and Negative (Underlined) Adjusted Standardised Residuals of Those Cells Greater Than 2 and Less Than −2, Respectively.

		Red	Orange	Yellow	Green	Aquamarine	Sky	Blue	Purple	Pink	Brown	White-Grey	Grey-Black
	Shape												
1	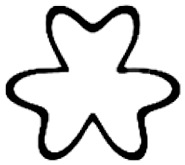	9.3	1.3	5.3	**37.3**	6.7	6.7	5.3	4.0	**20.0**	1.3	0	2.7
2	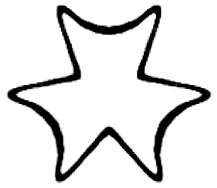	10.7	4.0	14.7	10.7	6.7	4.0	**16.0**	6.7	5.3	2.7	8.0	**10.7**
3	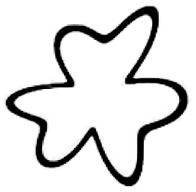	9.3	4.0	10.7	**24.0**	9.3	4.0	9.3	5.3	**21.3**	1.3	0	1.3
4	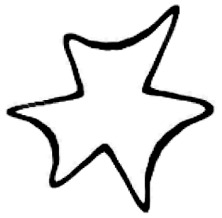	12.0	4.0	14.7	14.7	6.7	4.0	8.0	1.3	2.7	2.7	**20.0**	**9.3**
5	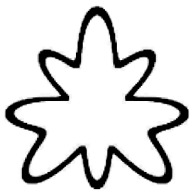	6.7	1.3	18.7	13.3	10.7	8.0	8.0	**14.7**	9.3	4.0	4.0	1.3
6	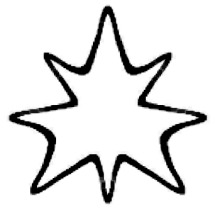	16.0	9.3	17.3	12.0	5.3	9.3	12.0	2.7	6.7	2.7	2.7	4.0
7	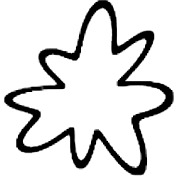	9.3	6.7	20.0	9.3	8.0	4.0	14.7	9.3	13.3	1.3	2.7	1.3
8	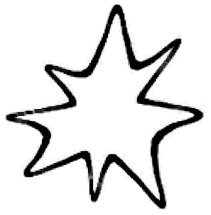	**21.3**	9.3	13.3	12.0	6.7	6.7	5.3	6.7	5.3	5.3	4.0	4.0
9	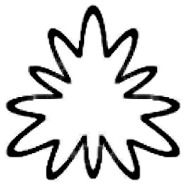	8.0	**12.0**	22.7	12.0	4.0	2.7	8.0	8.0	12.0	1.3	2.7	6.7
10	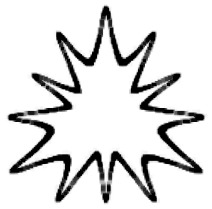	13.3	10.7	21.3	9.3	2.7	6.7	6.7	10.7	6.7	2.7	8.0	1.3
11	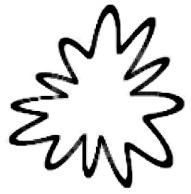	10.7	6.7	6.7	8.0	5.3	8.0	8.0	10.7	**21.3**	4.0	5.3	5.3
12	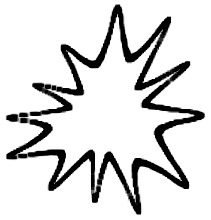	13.3	8.0	17.3	13.3	9.3	1.3	10.7	8.0	8.0	2.7	5.3	2.7
	Average	11.7	6.4	15.2	14.7	6.8	5.4	9.3	7.3	11.0	2.7	5.2	4.2

*Note*. The ‘average’ row represents the average percentage of individuals choosing each colour category across all shapes.

While there does appear to be a significant association between red and the circle (*z* = 2.0), as has been shown previously, the commonly found association between yellow and the triangle (Chen et al., 2014, 2015a, 2015c; Malfatti, 2014, published in Albertazzi et al., 2013) was not found. Furthermore, a larger residual was found for the circle and the yellow hue triplet (*z* = 2.9). Note that larger residuals, specifically those of 2 or more, can be interpreted as significantly larger cell counts than would be expected by chance, thus indicating that some form of nonrandom association exists between that specific shape and colour category, while negative residuals of −2 and less can be considered as significantly lower than expected, indicating that this colour category was chosen significantly less often than would have been expected if all colours were chosen with the same frequency.

While significant associations were found between specific shapes and colour hues in both Experiments 1 and 3, it is difficult with this type of analysis to make testable hypotheses for future research that extend beyond the specific stimuli sets used here. The reason for this being that each shape has its own set of perceptual characteristics that cannot readily be compared with another shape without further analysis and conceptualisation of each shape. As such, we must analyse both colour and shape in a way that allows us to examine the effect of perceivable colour and shape features.

### Colour and Shape Associations—Cluster Analyses by RGB Colour Characteristics

There are many ways of categorising and analysing colour and shape more broadly, as discussed earlier. Instead of dividing colours into categories determined by the experimenter's own understanding of them (or more accurately, the arbitrary divisions dictated by the need to create equal groups for statistical analysis), one could, for example, also use statistical methods to create clusters of colours. A two-step cluster analysis was conducted on 10 sets of the RGB colour values (RGB red [O], RGB green [O], RGB blue [O]) corresponding to each of the 36 colours (360 × 3 table). A two-step cluster analysis was performed on these 360 rows of data using Euclidean distances. This resulted in 6 clusters with a fair silhouette measure of cohesion and separation of 0.5. Table 4 shows what colours each cluster contains (for further information on cluster analysis, see Cluster Analysis: Basic Concepts and Algorithms, n.d.; Rousseeuw, 1987).

**Table 4.** Colours Contained in Each Cluster and Their Corresponding Mean RGB Values Obtained by a Two-Step Cluster Analysis of the RGB Data for all 36 Colours.

Cluster		RGB red mean	RGB green mean	RGB blue mean
1	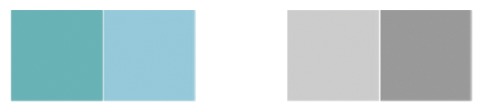	173.40	224.40	218.40
2	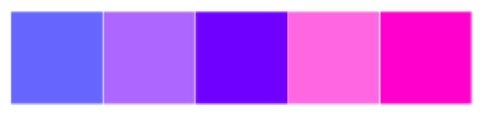	179.40	61.20	239.20
3	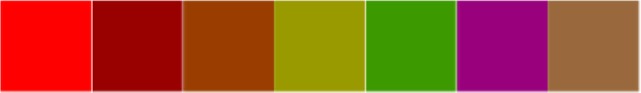 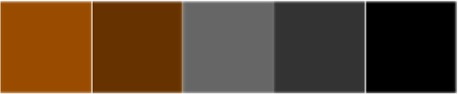	124.17	62.42	28.25
4	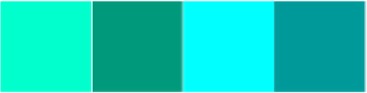	0.50	203.75	184.00
5	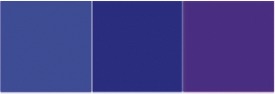	24.00	0.00	187.33
6	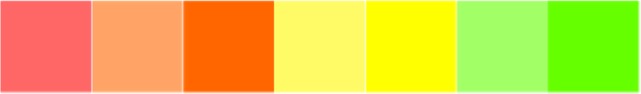	219.71	197.57	58.43

*Note*. These clusters were used to analyse all three experiments.

Instead of examining shape in terms of each individual shape as shown earlier, a multivariate analysis of variance (MANOVA) was conducted for each experiment to examine the differences in the case-by-case data of perceptually rated ([P]) colour characteristics of roundedness, symmetry, and complexity between the six clusters. In Experiment 1, there were significant differences in perceptual shape characteristics based on cluster membership, *F*(15, 10225) = 3.813, *p* < .0001; Wilk's λ = .985, partial η^2^ = .005. Statistically significant differences in roundedness, *F*(5, 3706) = 2.285, *p* = .044, partial η^2^ = .003; symmetry, *F*(5, 3706) = 4.185, *p* = .001, partial η^2^ = .006; and complexity, *F*(5, 3706) = 2.486, *p* = .030, partial η^2^ = .003 were found based on cluster membership. That is to say, the roundedness, symmetry, and complexity appear to have influenced what colour was chosen for a shape when colours are clustered into the six clusters outlined in Table 4. No significant differences were found between clusters in Experiment 2.

In Experiment 3, there were significant differences in perceptual shape characteristics ([P]) based on cluster membership, *F*(15, 2463) = 4.043, *p* < .001; Wilk's λ = .935, partial η^2^ = .022. Statistically significant differences in roundedness, *F*(5, 894) = 7.888, *p* < .0001, partial η^2^ = .042, and complexity, *F*(5, 894) = 3.695, *p* = .003, partial η^2^ = .020, were found based on cluster membership.

**Figure 6.**
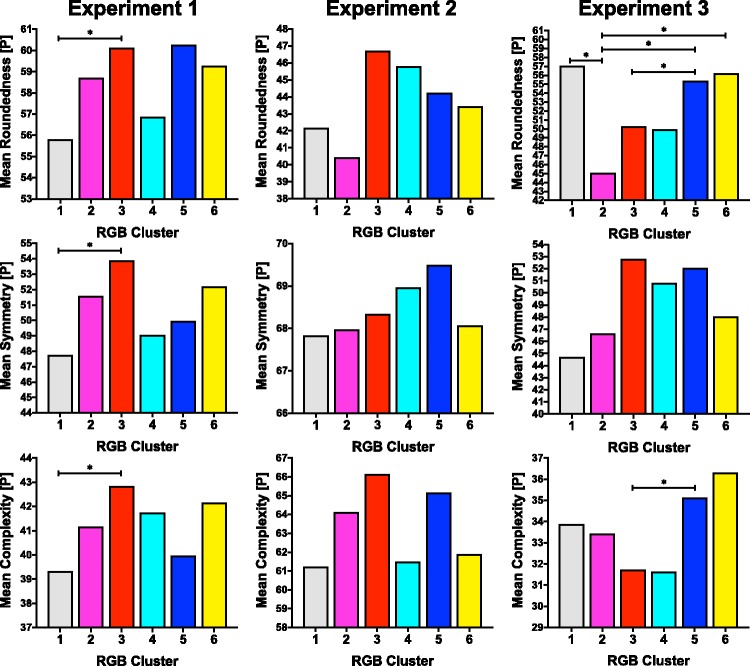
Tables showing the mean perceptual ([P]) roundedness, symmetry, and complexity for each colour cluster across the three experiments. Significant group differences (α = .05) are indicated with a bar and ‘*’.

Figure 6 shows the mean perceptual ([P]) roundedness, symmetry, and complexity for each cluster in each experiment as well as which group differences were found to be significant in Tukey post hoc tests. There are few, if any, discernible trends that are consistent across the three experiments for each shape characteristic.

### Colour and Shape Associations—MANOVAs of Case-By-Case Data

Another way in which to examine colour–shape associations is to examine the effect of each category level of shape characteristics on the perceptual colour characteristics for each colour chosen in the case-by-case data. To do this, a MANOVA was conducted for each experiment with the perceptual chosen colour characteristics as the dependent variable and the shape characteristic categories as the independent variables. The significant between-participant effects for each experiment are detailed in Tables 5 to 7.

**Table 5.** Significant Between-Participant Effects of Shape Categories on Perceptual Colour Characteristics for Case-By-Case Data in Experiment 1.

Source of variation from objective shape characteristics [O]	Dependent variable perceptual colour characteristics [P]	*F*	*df*	*p*
Symmetry	Green	4.907	1	.027
Roundedness	Red	3.454	2	.032
Concavities	Yellow	9.982	3	.000
	Blue	6.881	3	.000
	Saturation	6.308	3	.000
	Warmth	10.644	3	.000
Number of sides as levels	Yellow	5.217	3	.001
	Blue	4.497	3	.004
	Lightness	8.339	3	.000
	Saturation	6.136	3	.000
Symmetry × Number of Sides as Levels	Green	6.000	3	.000
	Yellow	2.670	3	.046
Roundedness × Concavities	Yellow	3.543	6	.002
	Lightness	2.699	6	.013
	Warmth	2.605	6	.016
Roundedness × Number of Sides as Levels	Yellow	2.792	4	.025
	Blue	2.946	4	.019
Symmetry × Roundedness × Concavities	Yellow	2.730	6	.012

**Table 6.** Significant Between-Participant Effects of Shape Categories on Perceptual Colour Characteristics for Case-By-Case Data in Experiment 2.

Source of variation from objective shape characteristics [O]	Dependent variable perceptual [P] colour characteristics	*F*	*df*	*p*
Roundedness	Lightness	7.679	1	.006
Complexity	Yellow-Blue	4.440	3	.004
	Yellow	4.385	3	.004
	Lightness	4.317	3	.005

**Table 7.** Significant Between-Participant Effects of Shape Categories on Perceptual Colour Characteristics for Case-By-Case Data in Experiment 3.

Source of variation from objective shape characteristics [O]	Dependent variable perceptual [P] colour characteristics	*F*	*df*	*p*
Symmetry	Lightness	4.705	1	.030
Roundedness	Red-Green	9.824	1	.002
	Red	9.743	1	.002
	Saturation	9.752	1	.002
Complexity	Yellow-Blue	3.632	2	.027
	Yellow	4.168	2	.016
	Lightness	3.451	2	.032
Symmetry × Complexity	Saturation	7.289	2	.001
Roundedness × Complexity	Green	3.613	2	.027

In Experiment 1, increased symmetry was associated with less greenness in the colours chosen. Angular and pointy shapes were less red than round shapes, and, overall, shapes with 1 concavity (vs. 0, 2, or 3 concavities) resulted in warmer, more yellow, less blue, and more saturated colours being chosen. Increasing the number of sides resulted in darker colour matches. Three-sided shapes were associated with the most saturated and bluest colour choices. Four-sided shapes were associated with lowest mean yellow colour choices, while one-sided shapes (i.e., variations on circles and ovals) were associated with more yellow colour choices. To be able to compare the number of sides with complexity [O], it should be noted that perceptual complexity increases with number of sides (1 side, mean = 11.67; 3 sides, mean = 20.32; 4 sides, mean = 29.87; 9 sides, mean = 63.56).

**Figure 7.**
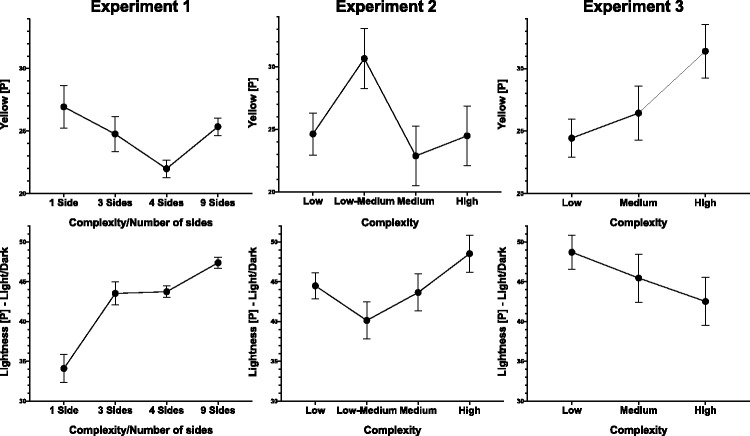
Graphs showing the average yellowness (first row) and lightness (second row) of colour choices at different levels of complexity [O] across the three experiments. In the case of Experiment 1, the number of sides [O] is taken as a proxy for complexity. The error bars represent the standard error of the mean.

In Experiment 2, rounded shapes (vs. pointy) are associated with lighter colours. Shapes in the low-medium complexity group had the highest levels of yellow, while more complex shapes were associated with darker colours. In Experiment 3, symmetrical shapes were associated with darker colours, while roundedness was associated with redder, less green, and less saturated colours. Increasing shape complexity was associated with more yellow, less blue, and lighter colour choices in Experiment 3 (see Figure 7).

The only consistent relationship across the three experiments appears to be an association between complexity with lightness [P] and yellow [P]. If we compare the number of sides as levels [O] in Experiment 1 (which have increasing complexity [P] with an increasing number of sides) with the effect of complexity [O] in Experiments 2 and 3 (see Figure 7), we can see, however, that this effect is not consistent in direction. Sometimes it is even completely opposite in direction, as is the case for lightness in Experiments 1 versus 3, where chosen colour lightness increases and decreases, respectively, with the increased complexity of the presented shape.

### Perceptual Colour and Shape Characteristics Associations—SCA Data

Another way to evaluate colour–shape associations by perceptual ratings is to look at correlations in the SCA data between perceptual colour and shape characteristics. As discussed earlier, this may be a better way in which to understand colour–shape perceptual associations than case-by-case data when looking at overall effects across the sample. The significant correlation results between perceptual shape and colour characteristics are presented in Table 8. It should be noted that no correlation is statistically significant consistently across the three experiments and that only the relationship between complexity and less blue colour choices survives a strict Bonferroni correction for multiple comparison. Furthermore, when compared with the ANOVAs conducted on the case-by-case data using category shape information, we see certain effects reflected in the SCA correlation results, but others are not borne out across the change in statistical test and shape feature. For example, the relationship between lightness and roundedness in Experiment 2 is found in both statistical tests, but the relationship of complexity with yellow and blue hues found in the ANOVAs is not found consistently in the perceptual SCA data. Therefore, by using a variety of tests, a more complex picture of colour–shape correspondences emerges.

**Table 8.** Significant Correlations in the SCA Data Between Perceptual Shape Characteristics ([P]) and Perceptual Colour Characteristics ([P]) Across the Three Experiments.

		Experiment 1	Experiment 2	Experiment 3
Roundedness	Red/Green	–	–	*r* = .592, *p* = .042
	Red	–	–	*r* = −.722, *p* = .008
	Green	*r* = −.293, *p* = .026	–	–
	Lightness	*r* = .265, *p* = .045	*r* = .500, *p* = .048	–
Symmetry	Green	*r* = −.275, *p* = .037	–	–
Complexity	Yellow/Blue	*r* = −.382, *p* = .003	–	*r* = −.670, *p* = .017
	Yellow	*r* = .277, *p* = .035	–	*r* = .756, *p* = .004
	Blue	*r* = −.464, *p* = .000	–	–
	Lightness	*r* = .304, *p* = .020	–	*r* = −.602, *p* = .038
	Warmth	*r* = .296, *p* = .024	–	–

SCA = shape–colour association

**Figure 8.**
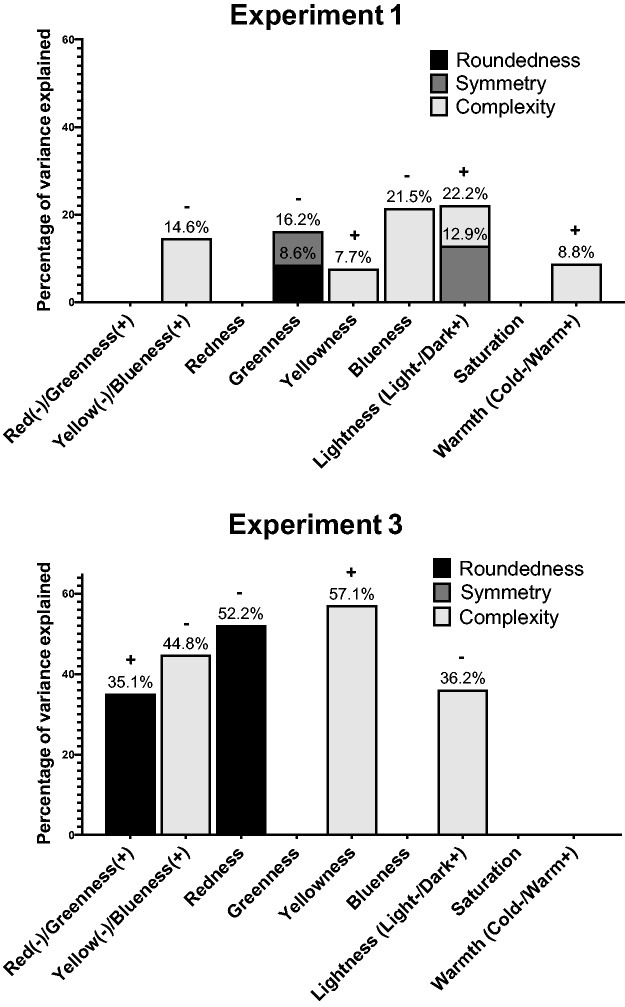
Multiple stepwise linear regression results for SCA data where perceptual shape characteristics predict perceptual colour characteristics for Experiments 1 and 3. The percentage of variance explained is annotated above each bar segment. The ‘+’ denotes a positive relationship and ‘−’ a negative relationship for all bar segments. Note that roundedness is measured on a scale from round (−) to pointy (+).

In Malfatti's (2014) study, the effect of the relationship of the perceptual shape dimensions on perceptual colour characteristics in the SCA data was examined using stepwise multiple linear regression. Replicating this methodology here, the SCA data are examined through stepwise multiple linear regressions, where each perceptual colour characteristic is entered as a dependent variable to be predicted by the perceptual shape characteristics of roundedness, symmetry, and complexity. No single relationship is found to be consistent across the three experiments (see Figure 8), although some relationships are consistent with the correlation findings shown in Table 8. The results of Experiment 2 are not presented because only 25% of the variance of lightness was predicted by roundedness, whereby the pointier a shape was, the darker the colour chosen. Furthermore, while the results of Experiment 3 in regard to the relationships between complexity–lightness and redness–roundedness are consistent with Malfatti's (2014, p. 82) results for closed geometric shapes, they are at odds with Malfatti's findings on saturation and yellow/blueness. Neither the results of Experiment 1 nor Experiment 2 are consistent with the results of Malfatti's SCA stepwise linear regression. On the other hand, the stepwise linear regression results for the SCA data are more in line with the case-by-case ANOVA results in regard to the association between complexity and yellow and blue hues than the SCA correlation data.

### Emotional Mediation Hypothesis Correlation Analyses—SCA Versus Case-By-Case Data

There are three ways in which we could think about liking and arousal appraisals having an effect on colour–shape correspondences. The first and most common way in which emotional mediation is examined in crossmodal correspondence research is to see whether there are significant correlations between the liking and arousal of a shape and the liking and arousal of the colour chosen for that shape (e.g., Velasco, Woods, Deroy, & Spence, 2015; Velasco et al., 2016; Wang, Wang, & Spence, 2016).

In Experiment 1, both on a case-by-case (see Table 9) and SCA data level (see Table 10), there is strong evidence for emotional mediation, although the correlation appears to be a lot stronger when considered through the aggregating lens of the SCA data. It should be noted that this emotional mediation does not only exist between matching appraisals but also between shape liking and chosen colour arousal (and vice versa). This is not so surprising when considering the strong correlation between shape liking and arousal as well as chosen colour liking and arousal.

**Table 9.** Pearson Correlation Matrix for Shape Liking and Arousal and the Liking and Arousal of the Colour Chosen for That Shape in Experiment 1 When Analysing Case-By-Case Data.

		Shape liking	Shape arousal	Chosen colour liking	Chosen colour arousal
Correlation	Shape liking	1			
	Shape arousal	.464**	1		
	Chosen colour liking	.190**	.108**	1	
	Chosen colour arousal	.139**	.163**	.479**	1

** Correlation is significant at the .01 level (two-tailed).

**Table 10.** Pearson Correlation Matrix for Shape Liking and Arousal and the Liking and Arousal of the Colour Chosen for That Shape in Experiment 1 When Analysing SCA Data.

		Shape liking	Shape arousal	Chosen colour liking	Chosen colour arousal
Correlation	Shape liking	1			
	Shape arousal	.824**	1		
	Chosen colour liking	.433**	.501**	1	
	Chosen colour arousal	.296*	.360**	.564**	1

*Note*. SCA = shape–colour association.

* Correlation is significant at the .05 level (two-tailed).

** Correlation is significant at the .01 level (two-tailed).

In Experiment 2, the statistically significant correlations between all variables are also found in the case-by-case data (see Table 11) as in Experiment 1, although the SCA data (see Table 12) in this case do not present as strong evidence as Experiment 1 for emotional mediation. Nevertheless, even in the SCA data, chosen colour liking is associated with both shape liking and arousal.

**Table 11.** Pearson Correlation Matrix for Shape Liking and Arousal and the Liking and Arousal of the Colour Chosen for That Shape in Experiment 2 When Analysing Case-By-Case Data.

		Shape liking	Shape arousal	Chosen colour liking	Chosen colour arousal
Correlation	Shape liking	1			
	Shape arousal	.495**	1		
	Chosen colour liking	.311**	.325**	1	
	Chosen colour arousal	.192**	.229**	.512**	1

** Correlation is significant at the .01 level (two-tailed).

**Table 12.** Pearson Correlation Matrix for Shape Liking and Arousal and the Liking and Arousal of the Colour Chosen for That Shape in Experiment 2 When Analysing SCA Data.

		Shape liking	Shape arousal	Chosen colour liking	Chosen colour arousal
Correlation	Shape liking	1			
	Shape arousal	.850**	1		
	Chosen colour liking	.547*	.506*	1	
	Chosen colour arousal	.302	.196	.267	1

*Note*. SCA = shape–colour association.

* Correlation is significant at the .05 level (two-tailed).

** Correlation is significant at the .01 level (two-tailed).

In Experiment 3, when we consider the case-by-case data (see Table 13), there is evidence for a statistically significant small to medium correlation between shape arousal and chosen colour arousal, while the other correlations, between shape liking and chosen colour liking, are very small, albeit statistically significant. The SCA data (see Table 14) reveal a large and statistically significant correlation between shape liking and chosen colour arousal, but all other emotional mediation effects are not statistically significant.

**Table 13.** Pearson Correlation Matrix for Shape Liking and Arousal and the Liking and Arousal of the Colour Chosen for That Shape in Experiment 3 When Analysing Case-By-Case Data.

		Shape liking	Shape arousal	Chosen colour liking	Chosen colour arousal
Correlation	Shape liking	1			
	Shape arousal	.250**	1		
	Chosen colour liking	.067*	.019	1	
	Chosen colour arousal	.069*	.247**	.494**	1

* Correlation is significant at the .05 level (two-tailed).

** Correlation is significant at the .01 level (two-tailed).

**Table 14.** Pearson Correlation Matrix for Shape Liking and Arousal and the Liking and Arousal of the Colour Chosen for That Shape in Experiment 3 When Analysing SCA Data.

		Shape liking	Shape arousal	Chosen colour liking	Chosen colour arousal
Correlation	Shape liking	1			
	Shape arousal	.856	1		
	Chosen colour liking	−.028	.150	1	
	Chosen colour arousal	.750**	−.064	.368	1

*Note*. SCA = shape–colour association.

* Correlation is significant at the .05 level (two-tailed).

** Correlation is significant at the .01 level (two-tailed).

In sum, then, the case-by-case data show more consistent evidence of emotional mediation effects, while the SCA does not have a specific pairing that is always significant but does overall also point towards the fact that individuals may be choosing colours based on the liking and arousal appraisals they make of both the shape and chosen colour. Arguably, it is the case-by-case data that better show whether an emotional mediation effect is taking place for this first type of emotional mediation because it reflects the matching behaviour of individuals at a choice-by-choice level. The SCA data on the other hand can show only emotional mediation effects if there is overall consistency between participants in terms of how colours and shapes are rated in terms of liking and arousal (i.e., if consensus exists concerning the extent and direction of liking and arousal for both shapes and colours). Where there is no consensus, the SCA is likely to show no emotional mediation effect. However, provided that each individual participant has more commonly matched colours and shapes emotionally congruent in terms of their own appraisals, the emotional mediation effect can still be picked up by case-by-case data analysis. As such, it is this type of data analysis that can more accurately answer whether individuals are matching colours and shapes based on hedonic appraisals.

A second way in which one can conceive of the emotional mediation of colour–shape matches is to consider whether shape appraisals have an effect on the types of colours that are chosen, as measured by their perceptual features. After correcting for multiple comparisons, the SCA data reveal that in Experiment 1 at least, shape arousal is associated with decreased green in colour choices (*r* = −.402, *p* = .002). This association between shape arousal and decreased greenness is also found in the case-by-case data (Red/Green: *r* = .108, *p* < .001, Green: *r* = −.059, *p* < .001) in Experiment 1. In Experiments 2 and 3, by contrast, none of the correlations remain significant in the SCA data after a Bonferroni correction for multiple comparisons.

The third way in which one can conceive of emotional mediation is to explore whether certain shape characteristics lead to the choice of colours that vary in terms of participants' liking and arousal ratings of them. After making a Bonferroni correction for multiple comparisons, in Experiment 1, there was a statistically significant medium-sized correlation between symmetry and chosen colour liking in Experiment 1 (*r* = .359, *p* = .006) found in the SCA data, which is also present in the case-by-case data (but which would not remain significant after correcting for multiple comparisons using a Bonferroni correction, *r* = −.043, *p* = .009). No significant correlations were found in the SCA and case-by-case data for Experiment 2. In Experiment 3, the SCA data revealed a large negative correlation between roundedness and chosen colour arousal (*r* = −.784, *p* = .003), which is also present in the case-by-case data (but would not remain significant after correcting for multiple comparisons using a Bonferroni correction, *r* = −.072, *p* = .030).

## Discussion

### Replicable Findings in the Context of Colour–Shape Correspondences Research

Consistent evidence for the existence of colour–shape correspondences could take two main forms. In the first instance, replicable findings would link a specific shape to a specific colour or set of hues across experiments. In the case of the three experiments reported here, only Experiments 1 and 3 revealed evidence of significant colour and shape associations of this type. It appears, then, to answer the first of the questions posed at the start of this article, that there can be colour–shape correspondences between specific shapes and hues for at least some shape stimuli. Whether these would be reliably replicated remains a question for future research. It should be noted, though, that even for the limited stimuli set of the Kandinsky shapes and colours (triangle, square, circle—red, blue, yellow), this type of evidence has not been consistent across experiments even if there do appear to be certain associations that are found more commonly than others for this particular set of stimuli (see earlier discussion and Figure 1; see Chapter 2 in Dreksler, 2019; Dreksler & Spence, 2019).

The second type of evidence for colour–shape correspondences would involve characteristics of a shape (e.g., roundedness) measured either perceptually or objectively, being associated with certain colour characteristics (e.g., redness). Even if specific colour–shape correspondences cannot be found reliably across experiments, such predictive associations may still exist, which is why we posed the second question earlier: Do specific shape characteristics predict the colours that are chosen, and do they do so reliably across stimuli sets and experiments? As predicted, it is certainly possible to find statistically significant and interesting trends of this nature within each colour–shape correspondence experiment. Significant results may not be found using each type of method of analysis, but within each experiment, certain shape characteristics were found to predict the nature of the colours chosen to best match the shapes. However, it is in the search for these types of colour–shape associations being replicable across stimuli that the current set of experiments has fallen short of finding evidence for. Therefore, to answer the second of the three questions posed at the start of this article, we do not find consistent evidence for specific shape characteristics predicting what colours are chosen across the three stimuli sets investigated using the same experimental design. While this may appear to constitute a blow to the notion that colour–shape correspondences exist, it is certainly not a surprising result in light of the mixed and complex nature of past research in this field. Of course, the field of research should remain open to accepting the null hypothesis that such replicable, consensual effects across cultural samples, experiments, and research groups may simply not exist. That being said, the results do show sufficient highly significant results and reasonable effect sizes that it seems inappropriate to accept the null hypothesis outright that this second type of colour–shape correspondence relationship does not exist at all (Frick, 1995). It may simply be that such relationships are highly stimuli dependent, depending both on the nature and range of stimuli used.

The final question we posed earlier is whether colour–shape matches are emotionally mediated. Indeed, there are reasonably consistent results across the three experiments indicating that whatever colour choices individuals may be making in terms of the perceptual colour features associated with them, they may in part be driven by the preference and arousal appraisals that individuals hold for colours and shapes. This is in line with an explanation in terms of the emotional mediation hypothesis (Palmer et al., 2013) that has been replicated in the case of colour–shape correspondences (Malfatti, 2014) and a variety of other crossmodal correspondences (e.g., Palmer et al., 2013; Schifferstein & Tanudjaja, 2004; Velasco et al., 2015; Wang et al., 2016), including colour-grapheme associations (Lau, Schloss, Eagleman, & Palmer, 2011; Simner et al., 2005). As such, it would seem reasonable to pursue and expand on how affective and semantic mechanisms may underlie colour–shape correspondences in future research.

### Potential Sources of Variation in Colour–Shape Correspondence Findings

The variation in SCAs found in the present study and past research could be caused by a variety of factors. At the sceptical end, we may argue that colour–shape correspondences are not a very strong, replicable, or dependable phenomenon and need to be discussed with more scrutiny by those researchers working in the field. Although the latter need is undoubtedly true, the lack of consistent results across the present three experiments could have been caused by the culturally diverse samples used. Much of the previous research has been conducted on university students with each research group presumably limiting themselves to participants from the same cultural background. This, arguably, could mean that individuals have more similar real-world associations with certain shapes, which could, in turn, result in more consistent colour–shape matching behaviour. As was discussed earlier, however, the results are not convincingly consistent even within such cultural samples. Therefore, other factors must be at play if we do not want to dismiss colour–shape correspondences outright. Furthermore, this explanation alone would speak against the universality of colour–shape correspondences, or at least that they are shared by a large number of people, which some have argued is a key feature of crossmodal correspondences (Spence, 2011).

In the case of these three experiments reported here, one may argue that the fact that the experiments were conducted online (adding some variation in colour lightness and hue through differences in monitors between participants) may have resulted in inconsistent results. While this may be true for the case-by-case data, the SCA data being an aggregate measure should be less affected by this potential confounding factor and yet shows similar inconsistencies across experiments. Finally, when considering Malfatti's (2014) results, which also showed different results between sets of shape stimuli, alongside these three experiments, it appears quite likely that colour–shape correspondences are a stimulus-dependent phenomenon. Each set of stimuli comes with its own set of appraisals and real-world, semantic, and emotional associations, which may be driving these inconsistencies. In addition, colour–shape correspondence research may simply not yet have found the right way in which to categorise, rate, and conceive of shape features that may be relevant to colour–shape correspondences. This lack of an ideal way of systematising shapes may be standing in the way of finding more consistent colour–shape correspondences.

A likely explanation is that a variety of the other factors detailed earlier contributed to the inconsistent results across experiments. In addition, colour–shape correspondence experiments may be examining a correspondence that is simply not a very strong phenomenon to begin with. Furthermore, the fact that many of the features of shape and colour that may be relevant for colour–shape correspondences are metathetic attributes, rather than more easily ordered prothetic dimensions, may further complicate the analysis and generalisability of colour–shape correspondence research. However, in contrast, crossmodal correspondences related to both colour and shape, including colour–taste and shape–taste correspondences, have been demonstrated reliably across many studies and decades of research (e.g., Spence et al., 2015; Velasco et al., 2016), so it is not the nature of colour and shape alone that can be blamed for the inconsistencies in colour–shape correspondence research (and indeed, as noted earlier, saturation and hue have been conceived as prothetic dimensions by some, see Gilbert et al., 2016; Panek & Stevens, 1966). With little research on intramodal correspondences having been published to date, it is difficult to assess whether the intramodal nature of colour–shape correspondences is the defining difference to the more reliably evidenced crossmodal correspondences related to colour and shape.

Overall, within the context of colour–shape correspondences, the many sources of variation, the evidence for it being a stimuli-dependent phenomenon, and the individual differences between participants that could play a role highlight the need for going beyond between-subjects designs and analyses and taking into account within-subject effects (for an in-depth look and comparison between both between- and within-subjects analyses for the earlier experiments and others, please see Chapter 3 of the doctoral dissertation Dreksler, 2019).

### Concluding Thoughts

If future researchers want to deepen our understanding of the nature of colour–shape correspondences and be able to find generalisable hypotheses about what shape characteristics are predictably associated with what colour features, especially when wider arrays of shapes and colours are presented to individuals, researchers will need to engage and wrestle with a number of issues:

1. Sources of variation: disentangling how the factors detailed earlier may affect colour–shape correspondences using both between-participant and within-participant designs.
2. Understanding of stimuli: search for new ways of conceiving of, and classifying, shapes and colours (including, e.g., other perceptual ratings but also potential objective methods, such as computational analyses of shapes).
3. Statistical analysis: consider how best to analyse colour–shape correspondences and consider evaluating them based not only on averages (SCA values) but also on a case-by-case basis using both between-participant and within-participant methods.
4. Critical review: present colour–shape correspondence research with the scrutiny it requires while casting a critical eye on the overall state of the field and the replicability of the findings therein.

While the field may yet be small and not be able to boast strong, replicable findings, it would also be incorrect to not further pursue the investigation of colour–shape correspondences or to write them off as a nonexistent phenomenon in the light of research conducted to date. Especially because intramodal correspondences remain an understudied field and colour and shape are commonly examined features in crossmodal correspondence research, colour–shape correspondences offer an excellent inlet into exploring the relationship between intramodal and crossmodal correspondences.

## Authors' Note

The research materials supporting this publication can be accessed by contacting noemi.dreksler@psy.ox.ac.uk. Please note that due to the confidential nature of some of the research materials supporting this publication not all of the data can be made accessible to other researchers.

## Notes

1. Colour temperature has been treated in colour–shape correspondences research as either a semantic concept without much elucidation (e.g., Chen et al., 2015d) or a simple division of the 40 NCS colour hue wheel into a warm half—yellowish greens, yellow, oranges, reds, and pinkish purples—and a cold half—greens, blueish greens, blue, and bluish purples (see Malfatti, 2014, p. 40).

2. Shape as a feature is therefore overall an amodal dimension but is here being assessed visually.

3. While colour–shape correspondences may be an intramodal correspondence (and shape is, strictly speaking, an amodal dimension), it stands to reason that similar mechanisms may underlie the existence of intramodal associations.

4. Arguably though, there is no strict differentiation between these emotional and nonemotional categories that Malfatti (2014) uses, and indeed, preference is considered elsewhere as a variable that falls under emotional mediation (Palmer et al., 2013).

5. Malfatti (2014) explored redness, greenness, yellowness, and blueness by asking individuals to rate them on scales of red-green and yellow-blue. We have chosen to use the same scales, for the sake of being able to compare our results with previous research, but have also included separate colour dimensions that range from ‘not at all' to ‘very' for each respective colour.

## References

[bibr1-2041669519834042] A new web-based colour vision test. (n.d.). Retrieved from https://www.city.ac.uk/health/research/centre-for-applied-vision-research/a-new-web-based-colour-vision-test.

[bibr2-2041669519834042] Albertazzi L. Da Pos O. Canal L. Micciolo R. Malfatti M. Vescovi M. (2013) The hue of shapes. Journal of Experimental Psychology: Human Perception and Performance 39: 37–47. doi:10.1037/a0028816. 22708741 10.1037/a0028816

[bibr3-2041669519834042] Albertazzi L. Malfatti M. Canal L. Micciolo R. (2015) The hue of angles—Was Kandinsky right?. Art & Perception 3: 81–92. doi:10.1163/22134913-00002025.

[bibr4-2041669519834042] Bagby R. M. Parker J. D. Taylor G. J. (1994) The twenty-item Toronto Alexithymia Scale—I. Item selection and cross-validation of the factor structure. Journal of Psychosomatic Research 38: 23–32. doi:10.1016/0022-3999(94)90005-1. 8126686 10.1016/0022-3999(94)90005-1

[bibr5-2041669519834042] Banaji M. R. Greenwald A. G. (2013) Blindspot: Hidden biases of good people, New York, NY: Random House.

[bibr6-2041669519834042] Baron-Cohen S. Wheelwright S. Skinner R. Martin J. Clubley E. (2001) The Autism-Spectrum Quotient (AQ): Evidence from Asperger syndrome/high-functioning autism, males and females, scientists and mathematicians. Journal of Autism and Developmental Disorders 31: 5–17. doi:10.1023/A:1005653411471. 11439754 10.1023/a:1005653411471

[bibr7-2041669519834042] Chen, N. (2015). *Semantic sensory correspondence between color and shape* (Doctoral dissertation). University of Tokyo, Japan. Retrieved from https://repository.dl.itc.u-tokyo.ac.jp/?action=repository_action_common_download&item_id=8283&item_no=1&attribute_id=14&file_no=1.

[bibr8-2041669519834042] Chen, N., Tanaka, K., Matsuyoshi, D., Nagamori, Y., Namatame, M., & Watanabe, K. (2014, January). *Color-shape association in deaf and hearing people*. Paper presented at the 6th International Conference on Knowledge and Smart Technology (KST), Chonburi, Thailand. doi:10.1109/kst.2014.6775405.

[bibr9-2041669519834042] Chen N. Tanaka K. Matsuyoshi D. Watanabe K. (2015a) Associations between color and shape in Japanese observers. Psychology of Aesthetics, Creativity, and the Arts 9: 101–110. doi:10.1037/a0038056.

[bibr10-2041669519834042] Chen N. Tanaka K. Matsuyoshi D. Watanabe K. (2015b) Cross preferences for colors and shapes. Color Research & Application 41: 188–195. doi:10.1002/col.21958.

[bibr11-2041669519834042] Chen, N., Tanaka, K., Namatame, M., & Watanabe, K. (2015c, January). *Consistency of color-shape associations in deaf people*. Paper presented at the 7th International Conference on Knowledge and Smart Technology (KST), Chonburi, Thailand. doi:10.1109/KST.2015.7051481.

[bibr122-2041669519834042] Chen, N., Tanaka, K., Namatame, M., & Watanabe, K. (2016). Color-shape associations in deaf and hearing people. *Frontiers in Psychology*, *7*, 355. doi:10.3389/fpsyg.2016.00355. 10.3389/fpsyg.2016.00355 PMC4791540 27014161

[bibr12-2041669519834042] Chen N. Tanaka K. Watanabe K. (2015d) Color-shape associations revealed with implicit association tests. PLoS One 10: e0116954 doi:10.1109/kst.2015.7051481. 25625717 10.1371/journal.pone.0116954 PMC4308101

[bibr13-2041669519834042] Chen Y.-C. Huang P.-C. Woods A. Spence C. (2016) When “Bouba” equals “Kiki”: Cultural commonalities and cultural differences in sound-shape correspondences. Scientific Reports 6: 26681 doi:10.1038/srep26681. 27230754 10.1038/srep26681 PMC4882484

[bibr14-2041669519834042] Cluster Analysis: Basic Concepts and Algorithms. (n.d.). Retrieved from https://www-users.cs.umn.edu/∼kumar001/dmbook/ch8.pdf.

[bibr15-2041669519834042] Dreksler, N. (2019). *Beyond Kandinsky: Exploring colour-shape correspondences through the lenses of emotions, individual differences and aesthetics* (Doctoral dissertation). Manuscript in preparation, University of Oxford, UK.

[bibr16-2041669519834042] Dreksler, N., & Spence, C. (2019). *Colour-shape correspondences: A historical primer and empirical literature review*. Manuscript submitted for publication.

[bibr17-2041669519834042] Ernst, M. O. (2007). Learning to integrate arbitrary signals from vision and touch. *Journal of Vision, 7*, 7.1–7.14. doi:10.1167/7.5.7. 10.1167/7.5.7 18217847

[bibr18-2041669519834042] Frick R. W. (1995) Accepting the null hypothesis. Memory & Cognition 23: 132–138. 7885262 10.3758/bf03210562

[bibr19-2041669519834042] Fryer L. Freeman J. Pring L. (2014) Touching words is not enough: How visual experience influences haptic-auditory associations in the “Bouba-Kiki” effect. Cognition 132: 164–173. doi:10.1016/j.cognition.2014.03.015. 24809744 10.1016/j.cognition.2014.03.015

[bibr20-2041669519834042] Gage J. (1993) Colour and culture: Practice and meaning from antiquity to abstraction, London, England: Thames & Hudson.

[bibr21-2041669519834042] Gilbert A. N. Fridlund A. J. Lucchina L. A. (2016) The color of emotion: A metric for implicit color associations. Food Quality and Preference 52: 203–210. doi:10.1016/j.foodqual.2016.04.007.

[bibr22-2041669519834042] Hanson-Vaux G. Crisinel A.-S. Spence C. (2012) Smelling shapes: Crossmodal correspondences between odors and shapes. Chemical Senses 38: 161–166. doi:10.1093/chemse/bjs087. 23118203 10.1093/chemse/bjs087

[bibr23-2041669519834042] Harrison J. E. (2001) Synaesthesia: The strangest thing, New York, NY: Oxford University Press.

[bibr24-2041669519834042] Henrich J. Heine S. J. Norenzayan A. (2010) The weirdest people in the world?. Behavioral and Brain Sciences 33: 61–83. doi:10.2139/ssrn.1601785. 20550733 10.1017/S0140525X0999152X

[bibr25-2041669519834042] Jacobsen T. (2002) Kandinsky's questionnaire revisited: Fundamental correspondence of basic colors and forms?. Perceptual and Motor Skills 95: 903–913. doi:10.2466/pms.95.7.903-913. 12509195 10.2466/pms.2002.95.3.903

[bibr26-2041669519834042] Jacobsen T. (2004) Kandinsky's color-form correspondence and the Bauhaus colors: An empirical view. Leonardo 37: 135–136.

[bibr27-2041669519834042] Jacobsen T. Wolsdorff C. (2007) Does history affect aesthetic preference? Kandinsky's teaching of colour-form correspondence, empirical aesthetics, and the Bauhaus. The Design Journal 10: 16–27. doi:10.1162/0024094041139193.

[bibr28-2041669519834042] John O. P. Naumann L. P. Soto C. J. (2008) Paradigm shift to the integrative big five trait taxonomy: History, measurement, and conceptual issues. In: John O. P. Robins R. W. Pervin L. A. (eds) Handbook of personality: Theory and research, New York, NY: Guilford Press, pp. 114–158.

[bibr29-2041669519834042] Just D. K. (2017) Was Kandinsky a synaesthete? Examining his writings and other evidence. Multisensory Research 30: 447–460. doi:10.1163/22134808-00002547. 10.1163/22134808-00002547 31287076

[bibr30-2041669519834042] Kandinsky W. (1914) The art of spiritual harmony, London, England: Constable and Co.

[bibr31-2041669519834042] Kandinsky, W. (1926/1994). *Point and line to plane* (P. Vergo, Trans.). In K. C. Lindsay & P. Vergo (Eds.), *Kandinsky, complete writings on art* (pp. 524–700). New York, NY: Da Capo Press.

[bibr32-2041669519834042] Kharkhurin A. V. (2012) Is triangle really yellow? An empirical investigation of Kandinsky's correspondence theory. Empirical Studies of the Arts 30: 167–182. doi:10.2190/em.30.2.d.

[bibr33-2041669519834042] Köhler W. (1929) Gestalt psychology, New York, NY: Liveright.

[bibr34-2041669519834042] Köhler W. (1947) Gestalt psychology, (2nd ed.) New York, NY: Liveright.

[bibr35-2041669519834042] Lau C. Schloss K. B. Eagleman D. M. Palmer S. E. (2011) Color-grapheme associations in non-synesthetes: Evidence of emotional mediation. Journal of Vision 11: 394 doi:10.1167/11.11.394.

[bibr36-2041669519834042] Lupton E. Miller J. A. (1993) The ABC's of triangle/square/circle: The Bauhaus and design theory, London, England: Thames and Hudson.

[bibr37-2041669519834042] Makin A. D. J. Wuerger S. (2013) The IAT shows no evidence for Kandinsky's color-shape associations. Frontiers in Psychology 4: 616 doi:10.3389/fpsyg.2013.00616. 24062709 10.3389/fpsyg.2013.00616 PMC3769683

[bibr38-2041669519834042] Malfatti, M. (2014). *Shape-to-color associations in non-synesthetes: Perceptual, emotional, and cognitive aspects* (Doctoral dissertation). University of Trento, Italy. Retrieved from http://eprints-phd.biblio.unitn.it/1336/.

[bibr39-2041669519834042] Marks D. F. (1973) Visual imagery differences in the recall of pictures. British Journal of Psychology 64: 17–24. doi:10.1111/j.2044-8295.1973.tb01322.x. 4742442 10.1111/j.2044-8295.1973.tb01322.x

[bibr40-2041669519834042] Maurer D. Pathman T. Mondloch C. J. (2006) The shape of boubas: Sound-shape correspondences in toddlers and adults. Developmental Science 9: 316–322. doi:10.1111/j.1467-7687.2006.00495.x. 16669803 10.1111/j.1467-7687.2006.00495.x

[bibr41-2041669519834042] Ngo M. K. Piqueras-Fiszman B. Spence C. (2012) On the colour and shape of still and sparkling water: Implications for product packaging. Food Quality & Preference 24: 260–268. doi:10.1016/j.foodqual.2011.11.004.

[bibr42-2041669519834042] Osgood C. E. Suci G. J. Tannenbaum P. H. (1957) The measurement of meaning, Urbana, IL: University of Illinois Press.

[bibr43-2041669519834042] Palmer S. E. Schloss K. B. Xu Z. Prado-León L. R. (2013) Music-color associations are mediated by emotion. Proceedings of the National Academy of Sciences of the USA 110: 8836–8841. doi:10.1073/pnas.1212562110. 23671106 10.1073/pnas.1212562110 PMC3670360

[bibr44-2041669519834042] Panek W. Stevens S. S. (1966) Saturation of red: A prothetic continuum. Perception & Psychophysics 1: 59–66. doi:10.3758/bf03207823.

[bibr45-2041669519834042] Parise C. V. Spence C. (2012) Audiovisual crossmodal correspondences and sound symbolism: A study using the implicit association test. Experimental Brain Research 220: 319–333. doi:10.1007/s00221-012-3140-6. 22706551 10.1007/s00221-012-3140-6

[bibr46-2041669519834042] Parise C. V. Spence C. (2013) Audiovisual crossmodal correspondences in the general population. In: Simner J. Hubbard E. M. (eds) The Oxford handbook of synesthesia, Oxford, England: Oxford University Press, pp. 790–815. doi:10.1093/oxfordhb/9780199603329.013.0039.

[bibr47-2041669519834042] Parise C. V. Spence C. Deroy O. (2016) Understanding the correspondences: Introduction to the special issue on crossmodal correspondences. Multisensory Research 29: 1–6. doi:10.1163/22134808-00002517. 27311288 10.1163/22134808-00002517

[bibr48-2041669519834042] Reinoso Carvalho F. Van Ee R. Rychtarikova M. Touhafi A. Steenhaut K. Persoone D. Leman M. (2015) Does music influence the multisensory tasting experience?. Journal of Sensory Studies 30: 404–412. doi:10.1111/joss.12168.

[bibr49-2041669519834042] Rothen N. Seth A. K. Witzel C. Ward J. (2013) Diagnosing synaesthesia with online colour pickers: Maximising sensitivity and specificity. Journal of Neuroscience Methods 215: 156–160. doi:10.1016/j.jneumeth.2013.02.009. 23458658 10.1016/j.jneumeth.2013.02.009

[bibr50-2041669519834042] Rousseeuw P. J. (1987) Silhouettes: A graphical aid to the interpretation and validation of cluster analysis. Journal of Computational and Applied Mathematics 20: 53–65. doi:10.1016/0377-0427(87)90125-7.

[bibr51-2041669519834042] Salgado-Montejo A. Alvarado J. A. Velasco C. Salgado C. J. Hasse K. Spence C. (2015) The sweetest thing: The influence of angularity, symmetry, and the number of elements on shape-valence and shape-taste matches. Frontiers in Psychology 6: 1382 doi:10.3389/fpsyg.2015.01382. 26441757 10.3389/fpsyg.2015.01382 PMC4569812

[bibr52-2041669519834042] Saluja S. Stevenson R. J. (2018) Cross-modal associations between real tastes and colors. Chemical Senses 43: 475–480. doi:10.1093/chemse/bjy033. 29868904 10.1093/chemse/bjy033

[bibr53-2041669519834042] Schifferstein H. N. J. Tanudjaja I. (2004) Visualizing fragrances through colors: The mediating role of emotions. Perception 33: 1249–1266. doi:10.1068/p5132. 15693669 10.1068/p5132

[bibr54-2041669519834042] Simner J. Ward J. Lanz M. Jansari A. Noonan K. Glover L. Oakley D. A. (2005) Non-random associations of graphemes to colours in synaesthetic and non-synaesthetic populations. Cognitive Neuropsychology 22: 1069–1085. doi:10.1080/02643290500200122. 21038290 10.1080/02643290500200122

[bibr55-2041669519834042] Spence C. (2011) Crossmodal correspondences: A tutorial review. Attention, Perception, & Psychophysics 73: 971–995. doi:10.3758/s13414-010-0073-7. 10.3758/s13414-010-0073-7 21264748

[bibr56-2041669519834042] Spence C. (2012) Managing sensory expectations concerning products and brands: Capitalizing on the potential of sound and shape symbolism. Journal of Consumer Psychology 22: 37–54. doi:10.1016/j.jcps.2011.09.004.

[bibr57-2041669519834042] Spence C. (2018) Crossmodal correspondences: A tutorial review. In: Howes D. (eds) Senses and sensation: Critical and primary sources Vol. III London, England: Bloomsbury Academic, pp. 91–125.

[bibr58-2041669519834042] Spence C. Wan X. Woods A. Velasco C. Deng J. Youssef J. Deroy O. (2015) On tasty colours and colourful tastes? Assessing, explaining, and utilizing crossmodal correspondences between colours and basic tastes. Flavour 4: 23 doi:10.1186/s13411-015-0033-1.

[bibr59-2041669519834042] Stevens S. S. (1957) On the psychophysical law. Psychological Review 64: 153–181. 13441853 10.1037/h0046162

[bibr60-2041669519834042] Turoman N. Velasco C. Chen Y. C. Huang P. C. Spence C. (2018) Symmetry and its role in the crossmodal correspondence between shape and taste. Attention, Perception, & Psychophysics 80: 738–751. doi:10.3758/s13414-017-1463-x. 10.3758/s13414-017-1463-x 29260503

[bibr61-2041669519834042] Velasco C. Woods A. Deroy O. Spence C. (2015) Hedonic mediation of the crossmodal correspondence between taste and shape. Food Quality & Preference 41: 151–158. doi:10.1016/j.foodqual.2014.11.010.

[bibr62-2041669519834042] Velasco C. Woods A. T. Petit O. Cheok A. D. Spence C. (2016) Crossmodal correspondences between taste and shape, and their implications for product packaging: A review. Food Quality and Preference 52: 17–26. doi:10.1016/j.foodqual.2016.03.005.

[bibr63-2041669519834042] Wang Q. J. Wang S. Spence C. (2016) “Turn up the taste”: Assessing the role of taste intensity and emotion in mediating crossmodal correspondences between basic tastes and pitch. Chemical Senses 41: 345–356. doi:10.1093/chemse/bjw007. 26873934 10.1093/chemse/bjw007 PMC4840871

[bibr64-2041669519834042] Witthoft N. Winawer J. Eagleman D. M. (2015) Prevalence of learned grapheme-color pairings in a large online sample of synesthetes. PLoS One 10: e0118996 doi:10.1371/journal.pone.0118996. 25739095 10.1371/journal.pone.0118996 PMC4349591

[bibr65-2041669519834042] Woods A. T. Velasco C. Levitan C. A. Wan X. Spence C. (2015) Conducting perception research over the internet: A tutorial review. PeerJ 3: e1058 doi:10.7287/peerj.preprints.921. 26244107 10.7717/peerj.1058 PMC4517966

