# CORRIGENDUM to A Critical Analysis of Colour–Shape Correspondences: Examining the Replicability of Colour–Shape Associations

**DOI:** 10.1177/2041669519884368

**Published:** 2019-10-15

Dreksler, N., & Spence, C. (2019). A critical analysis of colour—shape correspondences: examining the replicability of colour—shape associations. *i-Perception*, *10*(2), 1–34. doi: 10.1177/2041669519834042

The authors regret that in this article the SCA restructured data was said to derive from a methodology of analysis and is compared to results from Malfatti (2014). This is incorrect and based on a misunderstanding of the unit of analysis that was used in the dissertation by Malfatti. In the case of this article, the SCA data refers to data where the unit of analysis is the shape. The SCA data in Malfatti (2014) on the other hand refers to data where the unit of analysis is the participant. This should be taken into consideration when results are compared to the dissertation by Malfatti (2014), but does not change the internal comparisons made between the experiments for the SCA data. The authors apologise for their error.
